# Two new enzymes that liberate undecaprenyl-phosphate to replenish the carrier lipid pool during envelope stress

**DOI:** 10.1128/mbio.03710-24

**Published:** 2025-01-29

**Authors:** Ian J. Roney, David Z. Rudner

**Affiliations:** 1 Department of Microbiology, Harvard Medical School, Boston, Massachusetts, USA; National Institute of Child Health and Human Development (NICHD), Bethesda, Maryland, USA

**Keywords:** undecaprenyl-phosphate, C55-P, peptidoglycan, stress-response, SigM, bacterial cell envelope, WTA, PG

## Abstract

The 55-carbon isoprenoid, undecaprenyl-phosphate (UndP), is a universal carrier lipid that ferries most glycans and glycopolymers across the cytoplasmic membrane in bacteria. In addition to peptidoglycan precursors, UndP transports O-antigen, capsule, wall teichoic acids, and sugar modifications. How this shared but limited lipid is distributed among competing pathways is just beginning to be elucidated. We recently reported that in the bacterium *Bacillus subtilis*, the stress-response sigma factor SigM and its cognate anti-sigma factor complex respond to changes in the free UndP pool. When levels are low, SigM activates genes that increase flux through the essential cell wall synthesis pathway, promote the recycling of the lipid carrier, and liberate the carrier from other polymer pathways. Here, we report that two additional enzymes under SigM control help maintain the free pool of UndP. One, UshA (YqjL), resembles alpha-beta hydrolases and liberates UndP from undecaprenyl-monophosphate-linked sugars. The other, UpsH (YpbG), resembles metallophosphoesterases and releases UndP from undecaprenyl-diphosphate-linked wall teichoic acids polymers but not lipid-linked peptidoglycan precursors. UshA becomes critical for growth when UndP-linked sugars are sequestered, and the carrier lipid pool is depleted. Similarly, UpsH becomes essential for viability when UndPP-linked intermediates accumulate. Mutations in the predicted catalytic residues of both putative hydrolases abrogate their function arguing that they act directly to release UndP. These findings define two new enzymes that liberate the carrier lipid from UndP- and UndPP-linked intermediates and bolster the model that the SigM stress-response pathway maintains the UndP pool and prioritizes its use for peptidoglycan synthesis.

**IMPORTANCE:**

Motivated by the success of naturally occurring glycopeptide antibiotics like vancomycin, one arm of recent antibiotic discovery efforts has focused on compounds that bind lipid-linked precursors used to build extracytoplasmic polymers. Trapping these precursors depletes the universal carrier lipid undecaprenyl-phosphate, which is required for the synthesis of virtually all surface polymers, including peptidoglycan. Understanding how cells respond to this stress to restore the carrier lipid pool is critical to identifying effective drugs. Here, we report the identification of two new enzymes that are produced in response to the depletion of the carrier lipid pool. These enzymes recover the carrier lipid but cleave distinct lipid-linked precursors to do so.

## INTRODUCTION

Most bacteria use the 55-carbon isoprenoid, undecaprenyl phosphate (UndP), to transport sugars and glycopolymers across the cytoplasmic membrane (1). Arguably the most important among these is the monomeric building blocks of the cell wall peptidoglycan (PG) (2). The PG precursor, a disaccharide pentapeptide, is built on UndP in the cytoplasm. The undecaprenyl pyrophosphate (UndPP)-linked muropeptide, called lipid II, is then transported across the cytoplasmic membrane where it is polymerized and crosslinked into the PG meshwork. The byproduct of the polymerization reaction is UndPP, which is dephosphorylated by membrane phosphatases, and UndP is then flipped across the cytoplasmic membrane for reuse (3, 4). In addition to PG precursors, UndP transports O-antigen, capsule, exopolysaccharide, and secondary cell wall polymers like wall teichoic acids (WTAs) (1, 5). The carrier lipid is also used to ferry sugars across the membrane that are used to glycosylate surface polymers, proteins, and lipids. Despite its essential role in these diverse glycopolymer synthesis and modification pathways, UndP is kept at low levels in the cytoplasmic membrane (6). How cells distribute this limited resource among competing pathways is only beginning to be elucidated.

We have recently reported that in *Bacillus subtilis,* the alternative sigma factor, SigM, and its anti-sigma factor complex (YhdL-YhdK) function to prioritize UndP for cell wall synthesis (7). When the levels of free UndP are sufficiently high, the anti-sigma factor complex holds SigM inactive at the membrane. However, when levels of the carrier lipid are reduced, SigM is released and activates genes that increase PG synthesis, boost UndP recycling, and liberate the carrier lipid from non-essential surface polymer pathways. As part of our analysis of the SigM signaling pathway, we demonstrated that the sigma factor becomes essential when UndP-linked sugars or UndPP-linked intermediates are sequestered (7). Here, we investigated whether there are specific genes that are induced under SigM control that liberate the carrier lipid from these intermediates. Our analysis identified one gene, *ushA* (formerly *yqjL*), that is required for viability when UndP-sugars are trapped and a second gene, *upsH* (*ypbG*), that becomes essential when UndPP-linked secondary cell wall polymers build up. Our data suggest that UshA liberates UndP from UndP-linked sugars and UpsH liberates UndP from UndPP-linked precursors with the exception of lipid II. These findings provide further support for the model that the SigM stress-response pathway functions to prioritize the carrier lipid for peptidoglycan synthesis and suggest that analogous enzymes liberate UndP from intermediates in other bacteria.

## RESULTS

### YqjL is essential for growth when UndP-linked GlcNAc is sequestered

**Fig 1:**
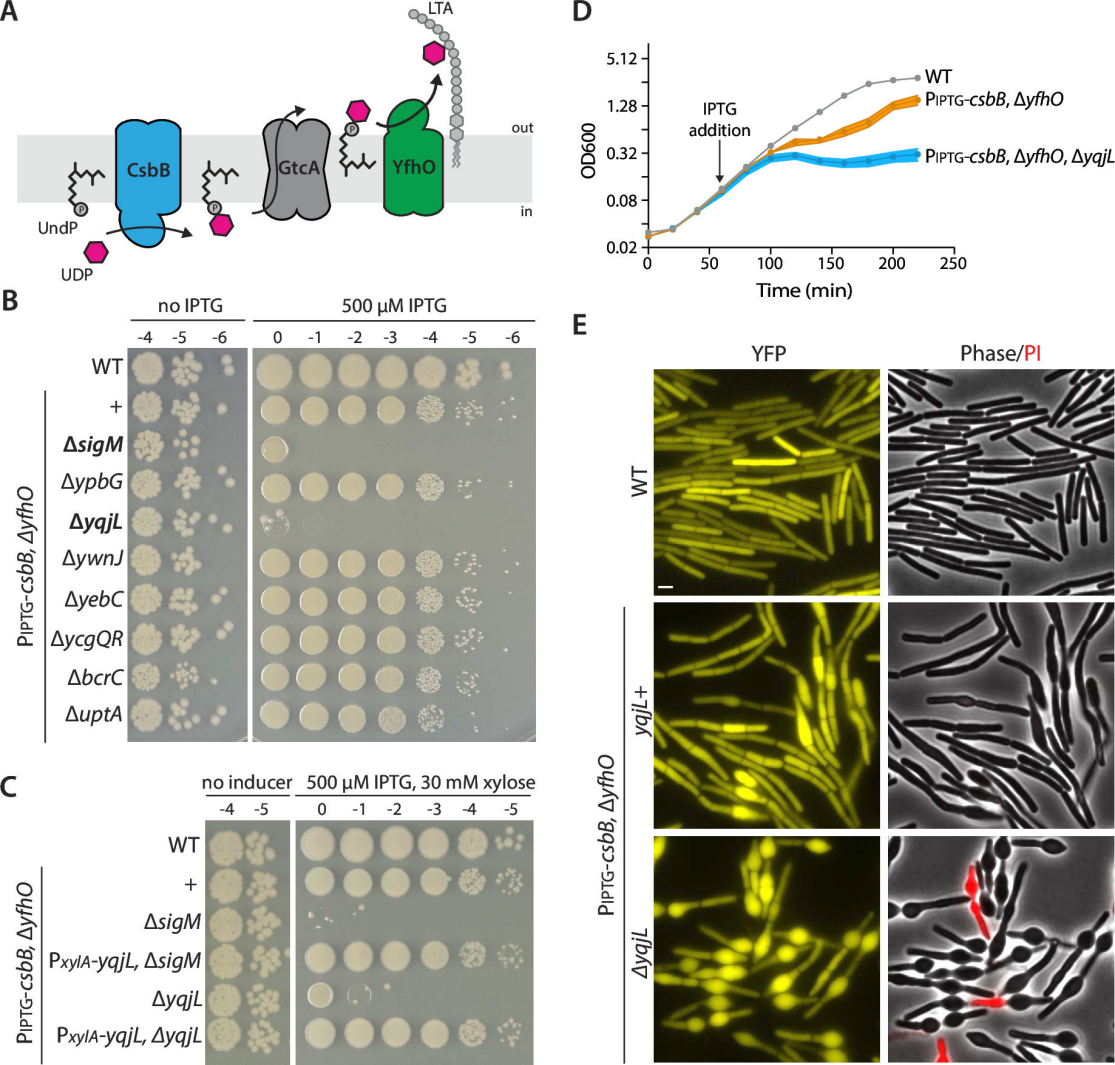
The SigM-regulated gene *yqjL* (*ushA*) becomes essential when UndP is trapped in the LTA glycosylation pathway. (**A**) Schematic diagram of the 3-protein LTA glycosylation pathway in *B. subtilis*. (**B, C**) Photographs of spot-dilution assays of the indicated *B. subtilis* strains grown on LB agar in the absence and presence of inducer. (**B**) Cells lacking *yqjL* (*ushA*) phenocopy the *sigM* mutant. (**C**) Expression of *yqjL* in *trans* restores growth of the ∆*yqjL* and ∆*sigM* mutants. (**D**) Growth curves of the indicated strains before and after the addition of IPTG (500 µM). (**E**) Representative phase-contrast and fluorescence images of the indicated strains 100 min after IPTG addition. Cytoplasmic YFP and phase-contrast highlight the bulged cell phenotype associated with defects in cell wall synthesis. Propidium Iodide (PI) reveals loss of membrane integrity. Scale bar indicates 2 µm.

Glycosylation of lipoteichoic acids (LTAs) on the outer leaflet of the cytoplasmic membrane is mediated by a 3-component pathway in *B. subtilis* (Fig. 1A) (8, 9). A glycosyltransferase, CsbB, generates UndP-GlcNAc on the cytoplasmic face of the membrane. The lipid-linked sugar is then flipped across the membrane by the GtcA transporter. Finally, the YfhO glycosyltransferase attaches GlcNAc onto the poly(glycerol-phosphate) chain of LTA. Overexpression of the committing enzyme, CsbB, in a strain lacking *yfhO* reduces the carrier lipid pool leading to SigM activation (7, 9). Under these conditions, SigM becomes essential for viability (7) (Fig. 1B). We hypothesized that a SigM-controlled gene was required to prevent sequestration of UndP-GlcNAc and the absence of SigM resulted in a lethal reduction in the UndP pool. To investigate this possibility, we tested a set of null mutations in genes of unknown function in the SigM regulon (Fig. 1B). We identified one gene (*yqjL*) with the expected phenotype. Deletion of *yqjL* phenocopied the ∆*sigM* mutation, with a 5-log plating defect when CsbB was overexpressed in the absence of *yfhO* (Fig. 1B). Expression of *yqjL* in *trans* suppressed the loss of viability (Fig. 1C). Furthermore, expression of *yqjL* from a xylose-regulated promoter restored viability to the strain lacking *sigM*, indicating that *yqjL* is the only SigM-controlled gene required to maintain viability when UndP-GlcNAc accumulates (Fig. 1C).

To explore the possibility that YqjL helps maintain the UndP pool when UndP-GlcNAc is trapped, we analyzed the morphology of ∆*yfhO* cells expressing CsbB in the presence and absence of *yqjL*. We induced *csbB* expression and analyzed the cells by fluorescence microscopy 100 min later, a time point when cell growth was already impaired (Fig. 1D). As can be seen in Fig. 1E, a subset of the *yqjL*+ cells displayed cell bulging. Importantly, these morphological defects were more severe and more prevalent in the ∆*yqjL* mutant, while overexpression of *yqjL* fully restored wild-type morphology (Fig. 1E; Fig. S1A). Cell bulging is a common phenotype of mutants with impaired cell wall synthesis (7), consistent with the sequestration of the carrier lipid and a drop in lipid II production. These results support the idea that YqjL helps maintain the free pool of UndP. Based on the experiments described below, we have renamed YqjL UshA for undecaprenyl-phosphate sugar hydrolase A.

### Evidence that UshA is an UndP-sugar hydrolase

**Fig 2:**
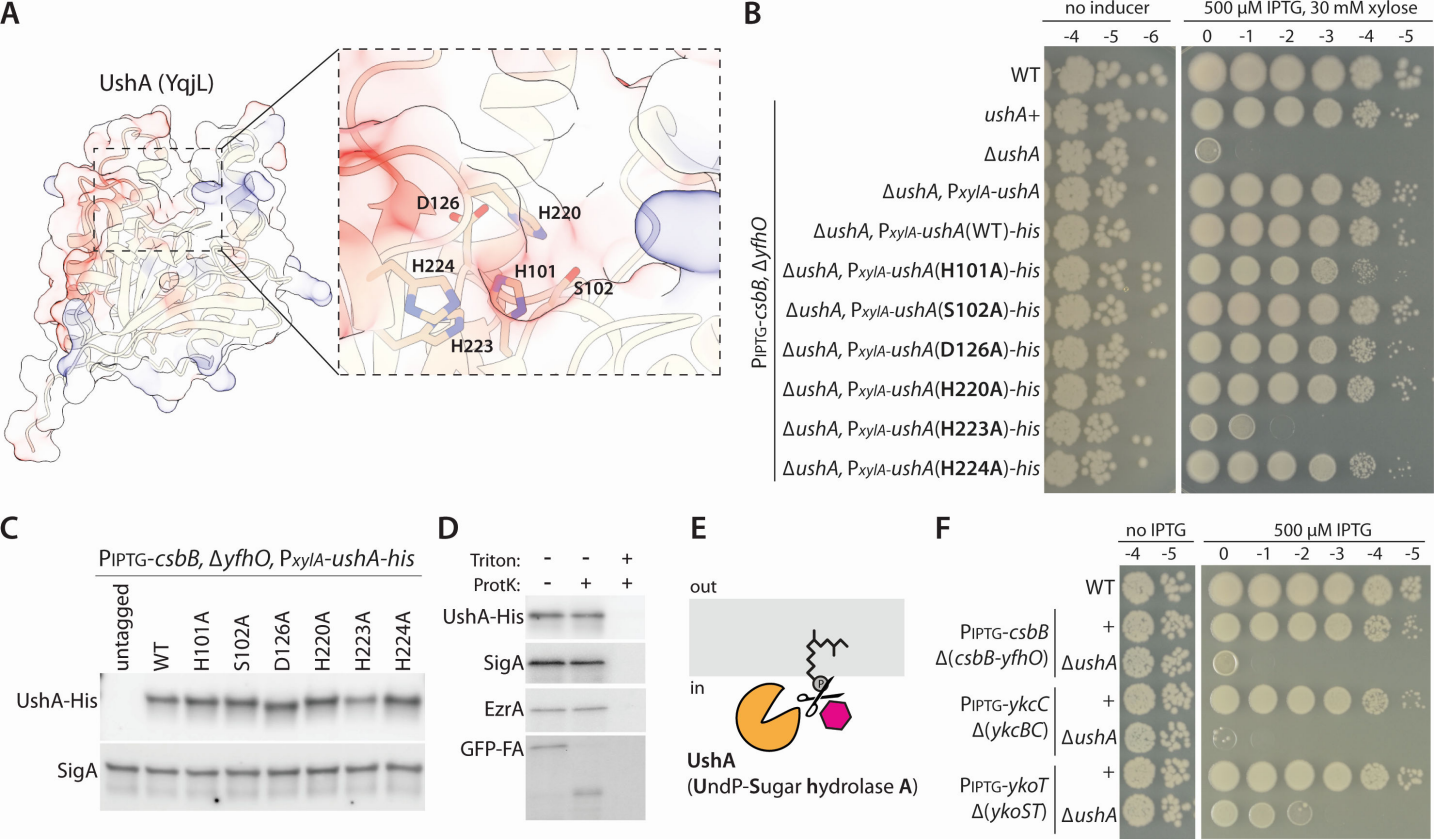
Mutations in the predicted catalytic pocket of UshA (YqjL) impair function. (**A**) AlphaFold-predicted structure of UshA with positive (blue) and negative (red) charged residues highlighted. The putative catalytic pocket is shown in greater detail with conserved residues indicated. (**B**) Photographs of spot-dilution assays of the indicated strains spotted on LB agar plates in the absence or presence of 500 µM IPTG and 30 mM xylose. Substitution of histidine 223 to alanine impairs UshA function. UshA(H101A) is modestly impaired. (**C**) Immunoblot analysis of UshA-His in the indicated strains. SigA controls for loading. (**D**) Representative immunoblots from a protease susceptibility assay. UshA-His, SigA, and EzrA are protected from Proteinase K (ProtK) cleavage in B. subtilis protoplasts. The extracytoplasmic domain of GFP-SpoIVFA (GFP-A) is susceptible. (**E**) Schematic diagram of proposed activity of UshA. (**F**) UshA is required for viability when UndP-linked sugar intermediates are trapped. Photographs of spot-dilution assays of the indicated *B. subtilis* strains grown on LB agar in the absence or presence of 500 µM IPTG.

Previous studies indicate that *ushA* is among the most highly induced genes in the SigM regulon (10). It encodes a putative alpha-beta hydrolase, suggesting it could cleave UndP-GlcNAc and liberate the carrier lipid. The alpha-beta hydrolase family is one of the largest groups of structurally related enzymes that catalyze diverse enzymatic reactions on a broad array of substrates, often acting at the membrane interface (11, 12). The UshA protein has a predicted catalytic pocket with several highly conserved residues (Fig. 2A; Fig. S2). To investigate whether these residues are important for function, we tested a set of alanine substitutions using a functional His-tagged UshA variant (Fig. 2B). Two of these mutants, H101A and H223A, were impaired in their ability to support growth under conditions in which UndP-GlcNAc accumulates (Fig. 2B). Importantly, these variants were expressed at levels similar to wild type (Fig. 2C). These data are consistent with the idea that UshA has enzymatic activity and functions to cleave UndP-GlcNAc to liberate UndP (Fig. 2D).

The UshA protein lacks a predicted signal peptide, suggesting it resides in the cytoplasm. To test this, we performed fractionation and protease accessibility assays using a strain that expresses the His-UshA fusion. The protein fractionated with soluble proteins (Fig. S3) and was protected from proteinase K degradation in protoplasts similar to the cytoplasmic protein SigA (Fig. 2E). By contrast, the GFP-SpoIVFA (GFP-FA) membrane protein that has an extracytoplasmic domain was protease accessible (13). We conclude UshA resides in the cytoplasm, and if it cleaves UndP-GlcNAc, it would do so before the lipid-linked sugar is transported across the membrane (Fig. 2D).

### UshA is required for viability when diverse undecaprenyl-monophosphate-linked sugars are sequestered

*B. subtilis* encodes two additional three-component surface polymer glycosylation pathways (8, 14). These pathways are less well characterized; the sugar modifications and their substrates are currently unknown. However, in both cases, the CsbB paralogs, YkcC and YkoT, are thought to generate UndP-linked sugars. Similarly, both pathways have glycosyltransferases of the GT-C superfamily (YkcB and YkoS) that are similar to YfhO and are likely involved in ligating the sugars onto LTA or some other extracytoplasmic glycopolymer (8, 14). We have recently shown that strains overexpressing YkcC or YkoT in the absence of their cognate transferase, YkcB or YkoS, activate SigM and require SigM for viability (7). To investigate whether UshA is responsible for maintaining viability under these conditions, we compared strains with and without *ushA*. As can be seen in Fig. 2F, cells lacking *ushA* were inviable or severely growth impaired when the committing glycosyltransferases were overexpressed in the absence of the cognate enzymes responsible for substate glycosylation. Collectively, these data suggest that UshA is capable of liberating UndP from diverse UndP-linked sugars.

### YpbG is essential for growth when UndPP-linked teichuronic acid intermediates are trapped

**Fig 3:**
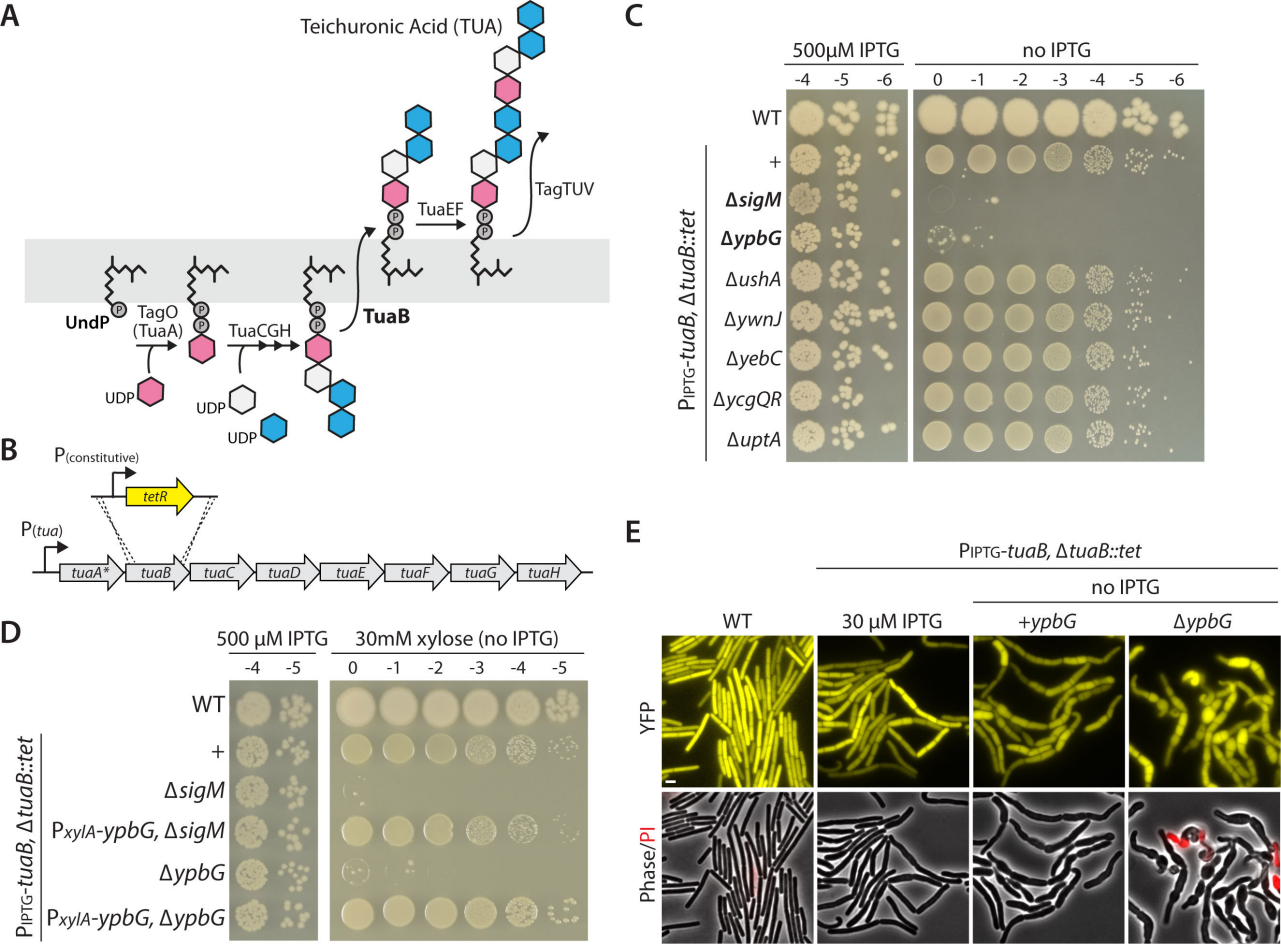
The SigM-regulated gene *ypbG* (*upsH*) becomes essential when UndP is trapped in the teichuronic acid biosynthesis pathway. (**A**) Schematic diagram of the teichuronic acid (TUA) biosynthesis pathway in *B. subtilis*. The *tuaA* gene is a psuedogene in the *B. subtilis* PY79 strain and TagO is thought to perform the committing step in its absence. The UndPP-linked precursor is flipped to the outer leaflet of the membrane by TuaB, shown in bold. (**B**) Schematic diagram of the *tua* operon. Insertional inactivation of *tuaB* with a tetracycline-resistance cassettes results in constitutive expression of the rest of the operon in phosphate replete medium, like LB. (**C, D**) Photographs of spot-dilution assays of the indicated *B. subtilis* strains grown on LB agar in the absence and presence of inducer. (**C**) Cells lacking *ypbG* (*upsH*) but not *ushA* phenocopy the *sigM* mutant. (**D**) Expression of *ypbG* in *trans* restores growth to the ∆*ypbG* and ∆*sigM* mutants. (**E**) Representative phase-contrast and fluorescence images of the indicated strains grown in the presence and absence of IPTG. YFP and phase-contrast highlight the bulged cell phenotype associated with defects in cell wall synthesis. Prodium iodide (PI) reveals membrane integrity defects. Scale bar indicates 2 µm.

In our characterization of the SigM signaling pathway, we also found that trapping undecaprenyl-diphosphate-linked precursors induces SigM-directed transcription and requires SigM for viability (7). Specifically, we found that overexpressing the teichuronic acid (TUA) biosynthesis operon while simultaneously reducing the levels of the putative flippase (TuaB) that transports UndPP-TUA precursors activated SigM (Fig. 3A and B) (7). Furthermore, reduced expression of TuaB was lethal in cells lacking SigM (Fig. 3C) (7). To investigate whether the expression of UshA was responsible for the loss of viability in the absence of SigM, we analyzed the ∆*ushA* mutant. Cells overexpressing the TUA biosynthesis operon with reduced expression of TuaB grew similarly in the presence and absence of *ushA* (Fig. 3C). These data suggest that UshA specifically acts on Und-monophosphate-linked sugars.

To explore the possibility that a distinct gene in the SigM regulon was required to maintain viability when Und-diphosphate-linked TUA precursors accumulate, we tested the same set of SigM-controlled genes that we used to identify *ushA*. A deletion of *ypbG* phenocopied the ∆*sigM* mutant, with a 6-log plating defect when TuaB levels were reduced (Fig. 3C). Expression of *ypbG* in *trans* suppressed the loss of viability (Fig. 3D). Furthermore, expression of *ypbG* from a xylose-regulated promoter restored viability to the strain lacking *sigM*, indicating that *ypbG* is the only SigM-controlled gene required to maintain viability when UndPP-TUA precursors accumulate (Fig. 3C). Finally, cells with trapped UndPP-TUA had morphological defects that were enhanced in the absence of *ypbG* (Fig. 3E) and overexpression of *ypbG* restored wild-type morphology (Fig. S1B).

We note that the gene encoding the committing enzyme for TUA synthesis, *tuaA*, is a pseudogene in domesticated *B. subtilis* strains like 168 and PY79. In these strains, TUA biogenesis is instead initiated by TagO, the committing enzyme in wall teichoic acid (WTA) synthesis (15). To exclude the possibility that the phenotypes we observed were due to the activity of a non-native committing enzyme, we expressed an intact *tuaA* gene in *trans*. Under these conditions, YpbG was also found to be critical for viability when TuaB levels were reduced (Fig. S4).

**Fig 4:**
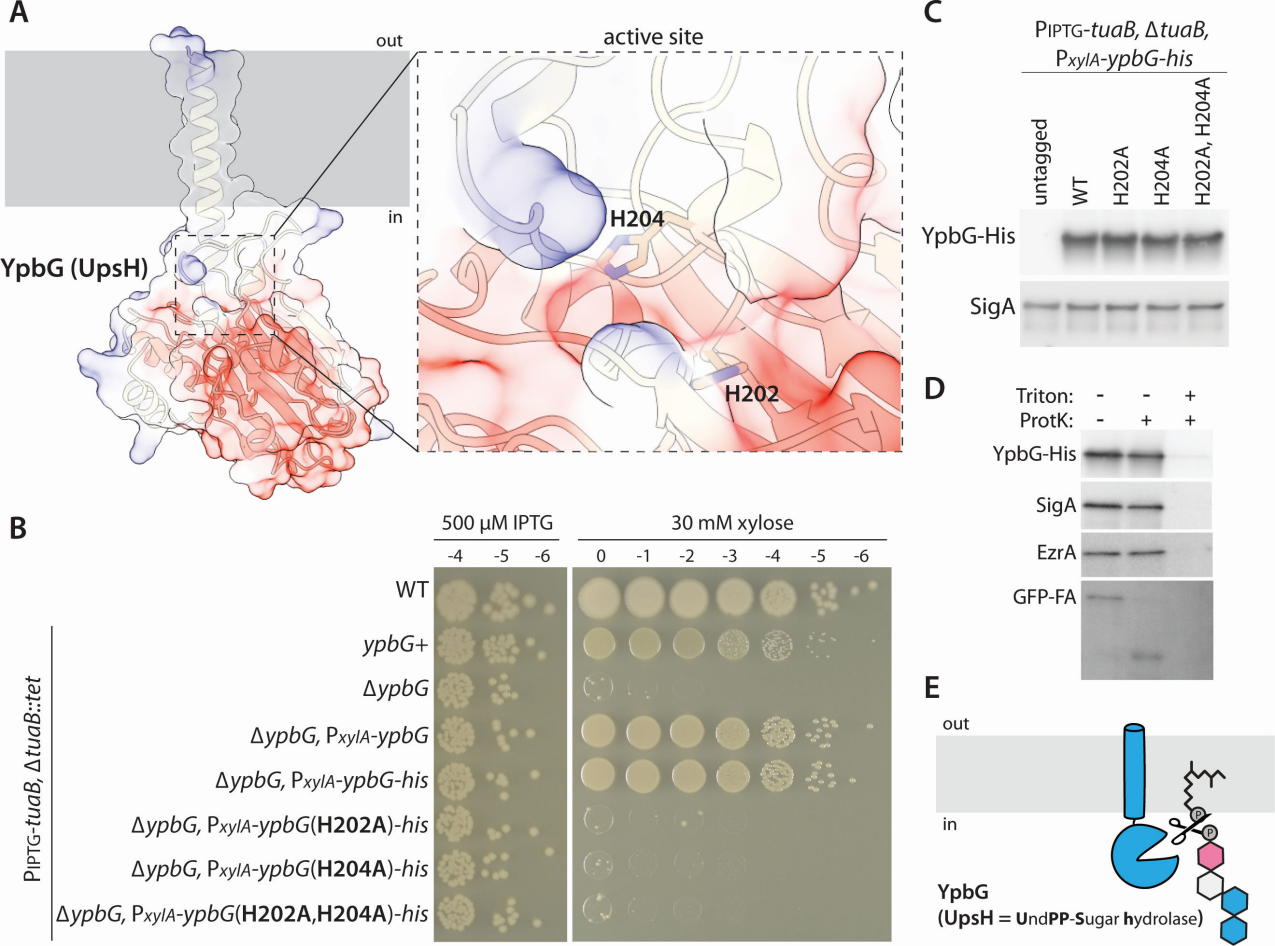
Mutations in the predicted catalytic histidines of YpbG (UpsH) impair function. (**A**) AlphaFold-predicted structure of YpbG with positive (blue) and negative (red) charged residues highlighted. The putative catalytic pocket is shown in greater detail with the highly conserved histidines indicated. (**B**) Photographs of spot-dilution assays of the indicated strains spotted on LB agar plates in the presence of 500 µM IPTG or 30 mM xylose. Substitutions of histidine 202 or 204 to alanine impair YpbG function. (**C**) Immunoblot analysis of YpbG-His in the indicated strains. SigA controls for loading. (**D**) Representative immunoblots from a protease susceptibility assay. YpbG-His, SigA, and EzrA are protected from Proteinase K (ProtK) cleavage in *B. subtilis* protoplasts. The extracytoplasmic domain of GFP-SpoIVFA (GFP-A) is susceptible. (**E**) Schematic diagram of the proposed activity of YpbG (UpsH) and its location in the inner leaflet of the cytoplasmic membrane.

Like *ushA*, *ypbG* is one of the most highly induced genes in the SigM regulon (10). It encodes a putative metallophosphoesterase (COG1408) (16), suggesting it could cleave UndPP-linked TUA precursors to liberate the carrier lipid. YpbG has a predicted catalytic pocket with two highly conserved histidines (H202 and H204) that in other metallophosphoesterases coordinate a metal ion and are required for phosphoesterase activity (Fig. 4A; Fig. S5). We tested alanine substitutions of these residues using a functional His-tagged YpbG fusion (Fig. 4B). Consistent with the idea that YpbG cleaves UndPP-TUA precursors, both mutants phenocopied the null (Fig. 4B). Moreover, the two point mutants were produced at levels similar to wild type (Fig. 4C).

YpbG is predicted to have a single transmembrane segment with its enzymatic domain located in the cytosol (Fig. 4A). The protein fractionated in both the soluble and membrane fractions (Fig. S3). Importantly, the His-YpbG fusion was not protease accessible in protoplasts, confirming that the enzymatic domain faces the cytoplasm (Fig. 4D). Based on these findings and those following, we have renamed YpbG UpsH for undecaprenyl-pyrophosphate sugar hydrolase (Fig. 4E).

### UpsH is required for viability when other undecaprenyl-pyrophosphate-linked secondary wall polymers are sequestered

**Fig 5:**
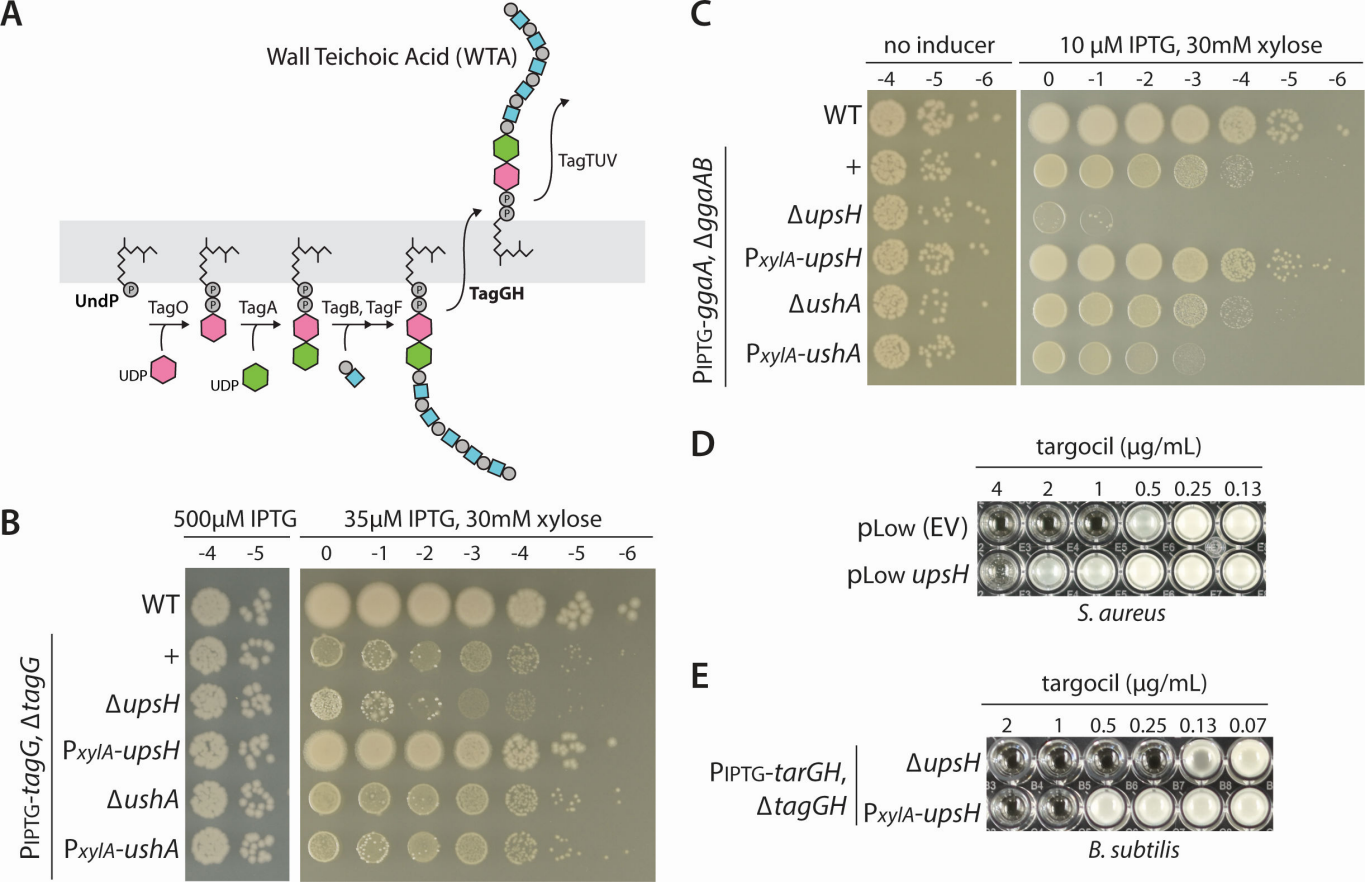
UpsH is required for viability when UndPP-linked WTA precursors are trapped. (**A**) Schematic diagram of the wall techioic acid (WTA) biosynthesis pathway in *B. subtilis*. The UndPP-linked WTA precursor is transported to the outer leaflet of the membrane by TagGH, shown in bold. (**B**) Photographs of spot-dilution assays of the indicated *B. subtilis* strains grown on LB agar in the presence of the indicated inducers. Low expression of TagG (35 µM IPTG) causes sequestration of UndPP-linked WTA precursors in the inner leaflet of the membrane, impairing growth. Under these conditions, *upsH* but not *ushA* becomes important for viability. Furthermore, overexpression of *upsH* but not *ushA* suppresses the growth defect. (**C**) Photographs of spot-dilution assays of the indicated *B. subtilis* strains grown on LB agar in the absence or presence of 10 µM IPTG and 30 mM xylose. Expression of *ggaA* in the absence *ggaB* traps an UndPP-linked intermediate in the minor WTA pathway. Low expression of *ggaA* with 10 µM IPTG impairs growth. Under this condition, *upsH* but not *ushA* becomes essential for viability and overexpression of *upsH* but not *ushA* suppresses the growth defect. (**D**) Photograph of wells from a 96-well plate used for Minimum Inhibitory Concentration (MIC) assays of the indicated *S. aureus* strains grown in TSB with twofold dilutions of targocil. Overexpression of *B. subtilis upsH* in *S. aureus* increases the MIC by 2- to 8-fold. (**E**) Photograph of wells from a 96-well plate used for MIC assays of the indicated *B. subtilis* strains grown in LB with twofold dilutions of targocil. The *B. subtilis* ∆*tagGH* mutant expressing of *S. aureus tarGH* is susceptible to targocil. The MIC of the ∆*upsH* mutant is 2- to 4-fold lower than a strain that overexpresses *upsH*. Images in panels D and E are from one of three biological replicates.

TUA is normally produced during phosphate limitation. It is a carbohydrate polymer containing glucuronic acid instead of poly(glycerol-phosphate) (17). To investigate whether UpsH maintains viability when phosphate-rich UndPP-linked major and minor teichoic acid precursors become trapped, we analyzed strains that accumulate UndPP-linked precursors. We took advantage of the observation that growth is impaired when the levels of the UndPP-linked WTA precursors build up due to reduction in the permease subunit (TagG) of the UndPP-WTA transporter (TagGH) (Fig. 5A) (18). Under these conditions, SigM-directed gene expression is induced (7). As can be seen in Fig. 4B, cells lacking *upsH,* but not *ushA,* have impaired growth when TagG levels are reduced. Even more strikingly, overexpression of UpsH (but not UshA) suppressed the growth defect resulting from the reduced expression of TagG (Fig. 5B). Next, we investigated whether UpsH helps maintain viability when UndPP-linked minor WTA intermediates accumulate. We have previously shown that overexpression of the committing enzyme (GgaA) in the synthesis of the minor teichoic acid in cells lacking the next enzyme (GgaB) in the pathway causes reduced growth and activation of SigM (7). As anticipated, the deletion of *upsH* (but not *ushA*) in this background resulted in the loss of viability (Fig. 5C). Growth inhibition was suppressed if UpsH was expressed in *trans*. Altogether, these data suggest that UpsH can liberate UndP from all known UndPP-linked secondary cell wall polymers.

In a complementary set of experiments, we took advantage of the antibiotic targocil (19) that inhibits the ABC transporter, TarGH, that flips UndPP-WTA to the outer leaflet of the cytoplasmic membrane in *Staphylococcus aureus* (18). Targocil kills *S. aureus* cells by trapping UndPP-WTA precursors in the inner leaflet of the membrane and depleting the carrier lipid pool. Figure 5D shows that expression of *B. subtilis* UpsH in *S. aureus* increased the minimum inhibitory concentration (MIC) of targocil by 2- to 8-fold compared to wild type. Thus, UpsH is sufficient to liberate UndP from the sequestered precursor, supporting the idea that UpsH catalyzes the reaction. Previous studies have shown that *B. subtilis* cells expressing TarGH*^Sa^* in the absence of the *B. subtilis* homologs (TagGH) are sensitive to targocil (18). Cells overexpressing UpsH in this strain background similarly increased the MIC of targocil 2- to 4-fold compared to a strain lacking *upsH* (Fig. 5E). Altogether, these data support the model that UpsH liberates UndP from UndPP-linked secondary wall polymers.

Finally, we investigated whether UpsH can act on the UndPP-linked PG precursor, lipid II. To do so, we used a strain in which the genes encoding the two lipid II flippases, *murJ* and *amj*, were deleted and contained an IPTG-regulated allele of *murJ* (7, 20). We analyzed growth over a range of IPTG concentrations in the presence and absence of *upsH* and when the protein was over-expressed (Fig. S6). All three strains were similarly growth impaired at low IPTG concentrations and healthy at high concentrations. We conclude that UpsH acts on UndPP-linked secondary wall polymers but not on the UndPP-linked PG precursor.

### **Expression of UshA and UpsH restores the carrier lipid pool when UndP(**P)-linked intermediates get trapped

**Fig 6:**
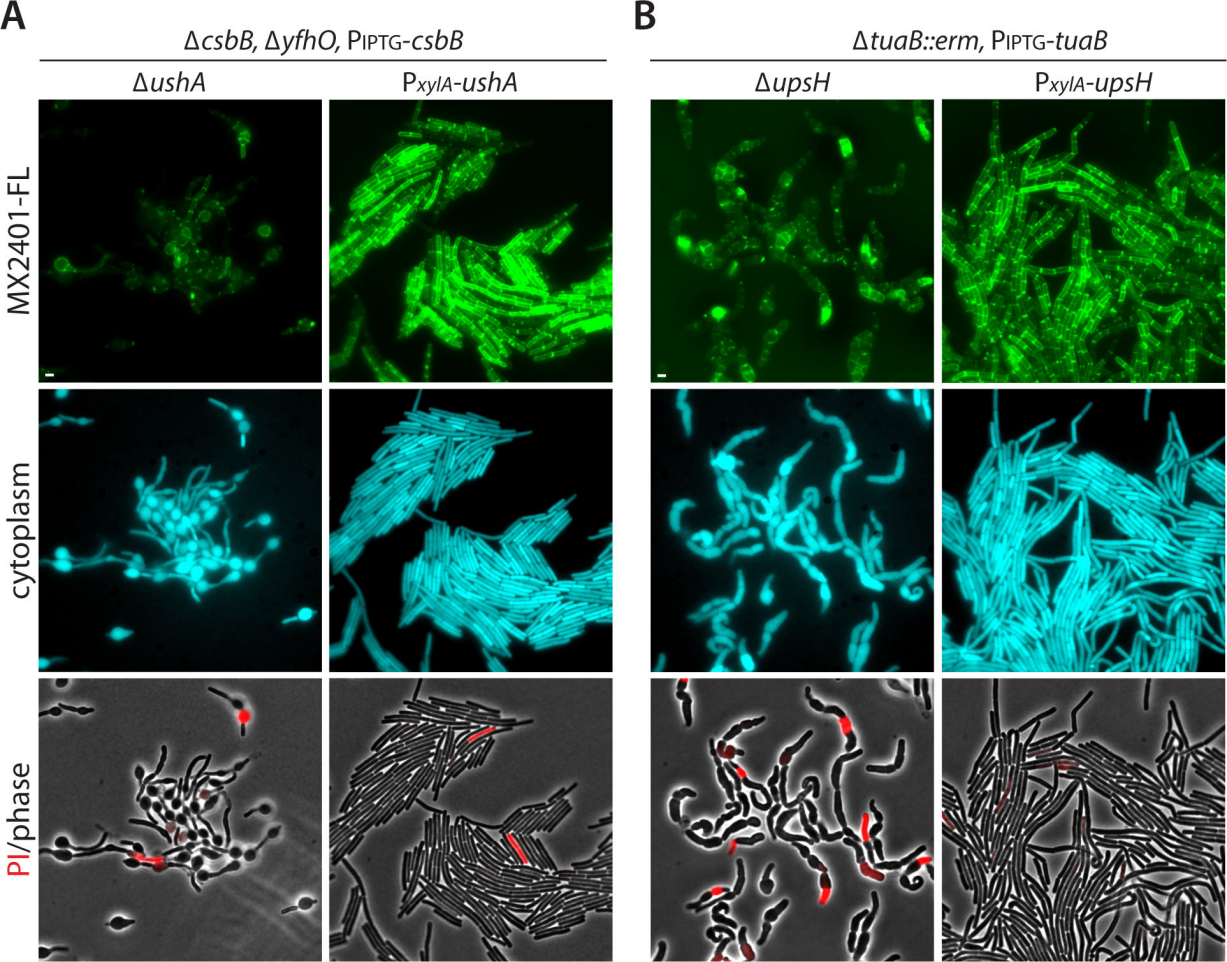
UshA and UpsH maintain the free pool of UndP. Representative fluorescence and phase-contrast images of strains stained with fluorescently labeled MX2401 (MX2401-FL) to assess the free pools of UndP. Expression of the blue fluorescent protein highlights the cell cytoplasm and phase-contrast merged with propdium iodide (PI) staining highlighting cells with membrane permeability defects. (**A**) Expression of CsbB in the absence of YfhO traps UndP-GlcNAc and in cells lacking UshA the carrier lipid pool is dramatically reduced. The reduction in UndP impairs cell wall synthesis resulting in aberrant morphologies. Overexpression of UshA increases the levels of UndP and restores normal cell shape. (**B**) Depletion of the TUA flippase TuaB traps UndPP-TUA precursors and in the absence of UpsH depletes the carrier lipid pool. Overexpression of UpsH increases the levels of UndP and restores normal cell morphologies. Scale bar indicates 2 µm.

Our data support a model in which UshA cleaves UndP-linked sugars to liberate UndP, while UpsH cleaves UndPP-linked precursors to return UndP to the carrier lipid pool. To further investigate this model, we used a fluorescently labeled antibiotic (MX2401) that binds the phosphate moiety on UndP (21). Using this probe (MX-FL), we monitored outward-facing UndP as a proxy for the carrier lipid pool (4, 7). First, we analyzed UndP levels in the ∆*yfhO* mutant overexpressing CsbB either in the absence of *ushA* or when UshA was overexpressed. After 90 min of CsbB expression in cells lacking UshA, the UndP pool was dramatically reduced (Fig. 6A). Consistent with a reduction in the carrier lipid pool, the cells had aberrant morphologies. By contrast, if UshA was overexpressed, the UndP pool remained high, and the morphological defects were largely suppressed (Fig. 6A). These data are consistent with the model that UshA liberates the UndP from UndP-GlcNAc restoring the carrier lipid pool.

In a parallel set of experiments, we compared UndP levels in strains depleted of TuaB that accumulate UndPP-linked TUA precursors. Cells lacking *upsH* had low levels of the carrier lipid as assayed by MX-FL and aberrant morphologies (Fig. 6B). However, the carrier lipid pool remained high, and the cells retained their normal rod shape if UpsH was overexpressed (Fig. 6B). These data support the model that UpsH cleaves UndPP-TUA liberating UndP.

## DISCUSSION

**Fig 7:**
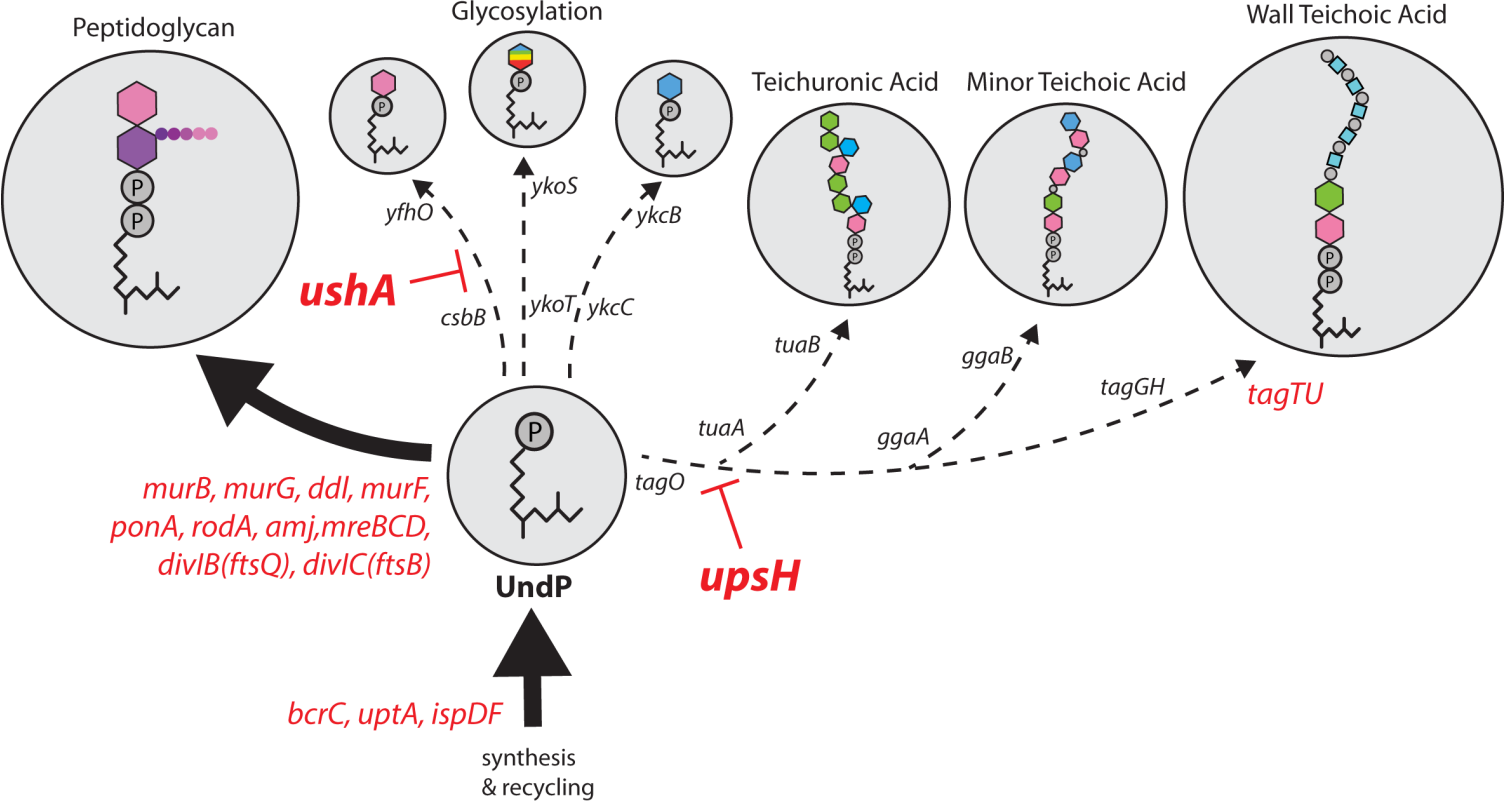
Schematic depiction of how SigM prioritizes cell wall synthesis and UndP synthesis and recycling when the carrier lipid pool drops. All the glycopolymer synthesis and modification pathways that use UndP are depicted. Genes controlled by SigM are highlighed in red. *murB*, *murG*, *ddl*, and *murF* are required for PG precursor synthesis; *amj* is required to transport the UndP-linked muropeptides (lipid II) across the cytoplasmic membrane; *ponA*, *rodA*, *mreBCD*, *divIB*, and *divIC* are required for PG synthesis. *bcrC* and *uptA* are required to recyle UndP and *ispD* and *ispF* are required for *de novo* synthesis of the carrier lipid. *tagT* and *tagU* are required to transfer UndPP-linked secondary cell wall polymers onto the PG, liberating UndP. *ushA* and *upsH* are required to liberate UndP from UndP-linked sugars and UndPP-linked second cell wall polymers, respectively. Collectively, in response to reduced levels of the carrier lipid, the SigM response increases flux through the PG biogenesis pathway and stimulates the liberation, recycling, and *de novo* synthesis of UndP.

Polyprenyl-phosphates are universal lipid carriers that transport sugars and glycopolymers across membranes in all domains of life (1, 5, 22, 23). In bacteria, UndP plays an essential role in transporting peptidoglycan precursors, teichoic acids, O-antigens of lipopolysaccharide, and capsule. The carrier lipid also transports sugars that are used to glycosylate surface polymers, lipids, and proteins. Despite its critical role in envelope biogenesis, UndP is maintained at low levels in the cytoplasmic membrane. In *B. subtilis*, the SigM-YhdLK stress-response pathway functions to prioritize this limited resource for the synthesis of the cell wall peptidoglycan (7). When levels of the carrier lipid are sufficiently high, SigM is held inactive at the membrane by YhdLK. However, when levels drop, SigM is released and activates a large set of genes that increase PG synthesis, boost UndP recycling, and liberate the lipid carrier from non-essential surface polymer pathways (Fig. 7). One mechanism of UndP liberation is through the SigM-controlled expression of two LCP phosphotransferases (TagT and TagU) that attach UndP-linked secondary cell wall polymers onto the PG, thereby releasing UndP (24, 25). Here, we provide evidence that SigM controls two additional enzymes that liberate the carrier lipid from UndP-linked sugars or -secondary cell wall precursors. Although we have not demonstrated enzymatic activity for either, both proteins are homologous to distinct hydrolases that catalyze similar reactions, the predicted catalytic residues of both proteins are essential for function, and in the case of UpsH, we have shown that this factor increases the MIC of targocil when expressed in *S. aureus*.

In both cases, these putative enzymes act on the cytoplasmic face of the lipid bilayer, prior to transport of the UndP-linked precursor. Accordingly, in addition to the carrier lipid, the attached sugars or polymers could be recycled for reuse. We speculate that homologs of UshA and UpsH function to liberate UndP in other organisms, and we predict that these enzymes are produced under the control of envelope stress-response transcription factors. Since most organisms encode multiple alpha-beta hydrolases and metallophosphoesterases (*B. subtilis* encodes 38 alpha-beta hydrolases and 18 metallophosphoesterases), the presence of these enzyme families in envelope stress-response regulons may be the easiest way to identify homologs.

Since SigM is active during unperturbed growth (26), we suspect it principally functions to homeostatically control UndP usage. Small changes in the free pool of carrier lipid likely cause small changes in the active pool of SigM and, in turn, tune the levels of the enzymes under its control. We hypothesize that these adjustments occur on the time-scale of minutes and, therefore, evolved to liberate UndP from substrates and precursors rather than adjusting the levels of the synthetic enzymes that produce these lipid-linked molecules. However, one could imagine an alternative strategy to control UndP usage in which the levels or activities of the committing enzymes in these non-essential pathways are regulated in response to UndP pools. In fact, recent work provides a precedent for this type of regulation. The *Listeria monocytogenes* eukaryotic-like serine/threonine membrane kinases PrkA was shown to control the degradation of MurA, the first committed step in PG precursor synthesis (27). We suspect other bacteria have evolved signaling pathways that employ phosphorylation or degradation to prioritize UndP usage, and we await the discovery of these pathways.

The SigM regulon has been a rich source of new enzymes that act on UndP and UndP-linked sugars. SigM controls the expression of an alternative lipid II flippase called Amj (20), presumably to increase flux of PG precursors when UndP levels are low. Similarly, RodA is under the control of SigM, and its presence in the regulon aided in the discovery that SEDS proteins are PG polymerases (28, 29). In addition, we recently reported that UptA is one of two UndP flippases that recycles the lipid carrier (3, 4). UptA is also under SigM control, and we suspect that increasing its expression ensures more rapid recycling and reuse of the carrier lipid. Here, we add two new factors under SigM control that liberate UndP. We hypothesize that there are still other genes of unknown function in the SigM regulon that increase flux through the PG biogenesis pathway, enhance UndP recycling, and liberate UndP from intermediates to replenish the carrier lipid pool.

## MATERIALS AND METHODS

### General methods

All *Bacillus subtilis* strains were derived from the prototrophic strain PY79 (30). All *B. subtilis* experiments were performed at 37°C with aeration in a defined casein hydrolysate (CH) medium or lysogeny broth (LB). Antibiotic concentrations used were 100 µg/mL spectinomycin, 10 µg/mL kanamycin, 5 µg/mL chloramphenicol, 10 µg/mL tetracycline, 1 µg/mL erythromycin, and 25 µg/mL lincomycin (MLS). All *B. subtilis* strains were generated using the one-step competence method unless indicated otherwise. All strains, plasmids, and oligonucleotides used in this study can be found in Tables S1 to S3.

### Statistics and reproducibility

All MIC experiments were performed in at least biological triplicate, and representative images are shown. All microscopy analyses were performed in at least biological duplicate with many fields of view analyzed, and representative images are shown. Spot assays and streak analysis were performed in at least biological triplicate. Western blotting was performed in biological duplicate. Attempts at replication for all experiments were successful.

### Spot-dilution assays

*B. subtilis* strains were grown at 37°C with aeration in lysogeny broth (LB) until cultures approached late-log phase. Cultures were normalized to OD600 = 1 and 10-fold serial dilutions were generated. Five microliters of each dilution was spotted onto LB agar supplemented with or without indicated concentrations of IPTG and/or xylose. Plates were incubated at 37°C overnight and photographed the next day.

### Minimal inhibitory concentration assays

Exponentially growing cultures of *B. subtilis* or *S. aureus* were back-diluted 1:10,000 into 96-well microtiter plates containing the indicated concentrations of antibiotic and inducers. Plates were sealed with breathable membranes and grown with orbital shaking at 37°C overnight. Plates were photographed after the overnight (~16 h) incubation. All MIC assays were performed in at least triplicate, and representative images are shown.

### Overexpression experiments

*B. subtilis* strains harboring IPTG-regulated alleles of *csbB*, *ykoS*, *ykoT, ggaA* were grown at 37°C with aeration in casein hydrolysate (CH) medium until cultures approached mid-log phase (OD600 = 0.2). IPTG was added to a final concentration of 500 µM, and samples were analyzed by fluorescence microscopy 100 min later.

### Depletion experiments

*B. subtilis* strains harboring IPTG-regulated alleles of *tuaB* or *tagG* were grown at 37°C with aeration in casein hydrolysate (CH) medium until cultures approached mid-log phase (OD600 = 0.5). The cells were visualized by fluorescence microscopy.

### MX2401-FL labeling

Exponentially growing cultures of *Bacillus subtilis* were collected by centrifugation at 7,000 RPM for 2 min. Cells were washed once with 1× PBS (pH 7.4) + 25 µg/mL CaCl_2_ and re-suspended in 1/25 volume of 1× PBS + 25 µg/mL CaCl_2_. MX2401 fluorescently labeled with CF488A (Biotium #92350) (MX2401-FL) (25 µM final) was added and incubated for 60 s. Cells were washed with 1× PBS, resuspended in 1/25 volume of 1× PBS, and spotted onto 1.5% agarose pads containing growth medium.

### Fluorescence microscopy

Cells were washed with 1× PBS, resuspended in 1/25 volume of 1× PBS, and spotted onto 1.5% agarose pads containing growth medium. Propidium iodide labeling was performed in 1× PBS at final concentrations of 5 µM.

Phase and fluorescence microscopy were performed with a Nikon Ti inverted microscope using a Plan Apo 100×/1.4 Oil Ph3 DM objective, a Lumencore SpectraX LED illumination system, and an Andor Zyla 4.2 Plus sCMOS camera. Chroma ET filter cubes (#49002, #49003, and 49008) were used for imaging MX2401-FL, YFP, and Propidium Iodide, respectively. Exposure time of 200 ms was used for MX2401-FL, 50 ms was used for propidium iodide, and 1 s was used for YFP. Images were acquired with Nikon elements 4.3 software and analyzed using ImageJ (version2.3).

### Immunoblot analysis

Immunoblot analysis was performed as described previously (31). Briefly, 1 mL of culture was collected and resuspended in lysis buffer (20 mM Tris [pH 7.0], 10 mM MgCl_2_ and 1 mM EDTA, 1 mg/mL lysozyme, 10 µg/mL DNase I, 100 µg/mL RNase A, 1 mM PMSF, 1 µg/mL leupeptin, 1 µg/mL pepstatin) to a final OD_600_ of 10 for equivalent loading. The cells were incubated at 37°C for 10 min followed by the addition of an equal volume of sodium dodecyl sulfate (SDS) sample buffer (0.25 M Tris [pH 6.8], 4% SDS, 20% glycerol, 10 mM EDTA) containing 10% 2-mercaptoethanol. Samples were heated for 15 min at 65°C prior to loading. Proteins were separated by SDS-PAGE on 12.5% polyacrylamide gels, electroblotted onto Immobilon-P membranes (Millipore), and blocked in 5% nonfat milk in phosphate-buffered saline (PBS) with 0.5% Tween-20. The blocked membranes were probed with anti-EzrA (1:10,000) (32), anti-SpoIVFA (1:10,000) (33), anti-SMC (1:10,000) (34), anti-SigA (1:10,000) (35), anti-GFP (1:10,000) (33) diluted into 3% BSA in 1× PBS with 0.05% Tween-20. Primary antibodies were detected using IRDye 800CW goat anti-rabbit (1:10,000) (Li-Cor) or IRDye 680RD goat anti-mouse (1:10,000) (Li-Cor). Signal was detected using a Bio-Rad ChemiDoc MP Imaging System.

### Fractionation

Twenty-five milliliters of exponentially growing cells was collected, washed, and resuspended in 5 mL 1× SMM buffer (0.5 M sucrose, 20 mM MgCl_2_, 20 mM maleic acid, pH 6.5) (36) supplemented with lysozyme (4 mg/mL). Cells were incubated for 30 min at RT with gentle agitation. Protoplast formation was monitored by microscopic observation on 2% 1× SMM-agarose pads. When >95% of the cells were protoplasted, they were collected by centrifugation (5 krpm) flash-frozen in N2(l). Thawed protoplasts were disrupted by osmotic lysis with 3 mL hypotonic buffer (buffer H) (20 mM HEPES [pH 8], 100 mM NaCl, 1 mM DTT, with protease inhibitors: 1 mM PMSF, 0.5 µg/mL leupeptin, 0.7 µg/mL pepstatin). MgCl_2_ and CaCl_2_ were added to 1 mM, and lysates were treated with DNase I (10 µg/mL) (Worthington) and RNase A (20 µg/mL) (USB) for 1 h on ice. A sample was removed (lysate) and mixed with 2× sample buffer (0.25 M Tris [pH 6.8], 4% SDS, 20% glycerol, 10 mM EDTA 10% 2-mercaptoethanol). The rest was subject to ultracentrifugation at 100,000 × *g* for 1 h at 4°C. The supernatant (S100) was carefully removed, and a sample was removed and mixed with 2× sample buffer. The pellet was dispersed in an equal volume of buffer H using a teflon dounce homogenizer, and then a sample was removed (P100) and mixed with 2× sample buffer. The samples were subject to SDS-PAGE and immunoblot analysis as described above.

### Protease accessibility assay

Twenty-five milliliters of exponentially growing cells was collected, washed, and resuspended in 5 mL 1× SMM buffer (0.5 M sucrose, 20 mM MgCl_2_, 20 mM maleic acid, pH 6.5) (36) supplemented with lysozyme (4 mg/mL). Cells were incubated for 30 min at RT with gentle agitation. Protoplast formation was monitored by microscopic observation on 2% 1× SMM-agarose pads. When >95% of the cells were protoplasted, they were collected by centrifugation (5 krpm) and resuspended in 1 mL of 1× SMM buffer and distributed into three microfuge tubes. Protoplasts were incubated with 1× SMM buffer alone or with proteinase K (NEB, 50 µg/mL final) or proteinase K and Triton X-100 (1%) for 15 min at room temperature. Proteinase K was inactivated by the addition of 2× sample buffer (0.25 M Tris [pH 6.8], 4% SDS, 20% glycerol, 10 mM EDTA 10% 2-mercaptoethanol) supplemented with 2 mM PMSF and immediately incubated at 100°C for 10 min. Reactions were analyzed by immunoblot.

### WebLogo and structural model generation

WebLogos (37) and ColabFold structural models (38) were generated using the ConservFold server (39) (accessible at https://www.rodrigueslab.com/resources).

### Structural model visualization

ChimeraX1.3 was used to visualize the structural models and generate images. Residues forming the putative catalytic clefts are shown as sticks. Structural models are colored according to conservation with blue indicating non-conserved residues and red indicating conserved residues.

## SUPPLEMENTAL MATERIAL

Supplemental Material mbio.03710-24-s0001.pdf Supplemental methods, figures, and tables.

ASM does not own the copyrights to Supplemental Material that may be linked to, or accessed through, an article. The authors have granted ASM a non-exclusive, world-wide license to publish the Supplemental Material files. Please contact the corresponding author directly for reuse.
