# Supplementary material for: Two new enzymes that liberate undecaprenyl-phosphate to replenish the carrier lipid pool during envelope stress
Source: mBio. 2025 Jan 29;16(3):e03710-24. doi: 10.1128/mbio.03710-24 (PMC11898649; doi:10.1128/mbio.03710-24)
Supplement: Supplemental Material — Supplemental methods, figures, and tables. [file mbio.03710-24-s0001.pdf]

Title: Two new enzymes that liberate undecaprenyl-phosphate to replenish the carrier lipid pool during envelope stress

Authors: Ian J. Roney and David Z. Rudner

Supplemental Material Includes:

Supplemental Figures (S1 – S6)
Supplemental Methods
Supplemental Tables (S1 – S3)

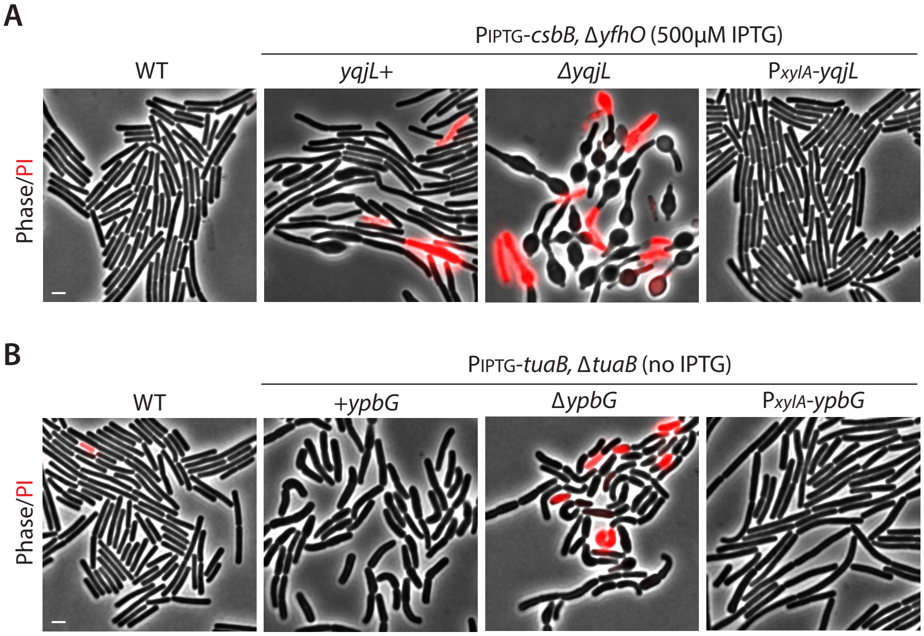

Figure S1. (A) Representative phase-contrast images of the indicated strains 90 min after IPTG addition. Phase-contrast highlight the bulged cell phenotype associated with defects in cell wall synthesis. Propidium Iodide (PI) reveals loss of membrane integrity. **(B)** Representative phase-contrast images of the indicated strains 90 min after withdrawal of IPTG. Phase-contrast highlight the bulged cell phenotype associated with defects in cell wall synthesis. Propidium Iodide (PI) reveals loss of membrane integrity. Scale bar indicates 2 µm.

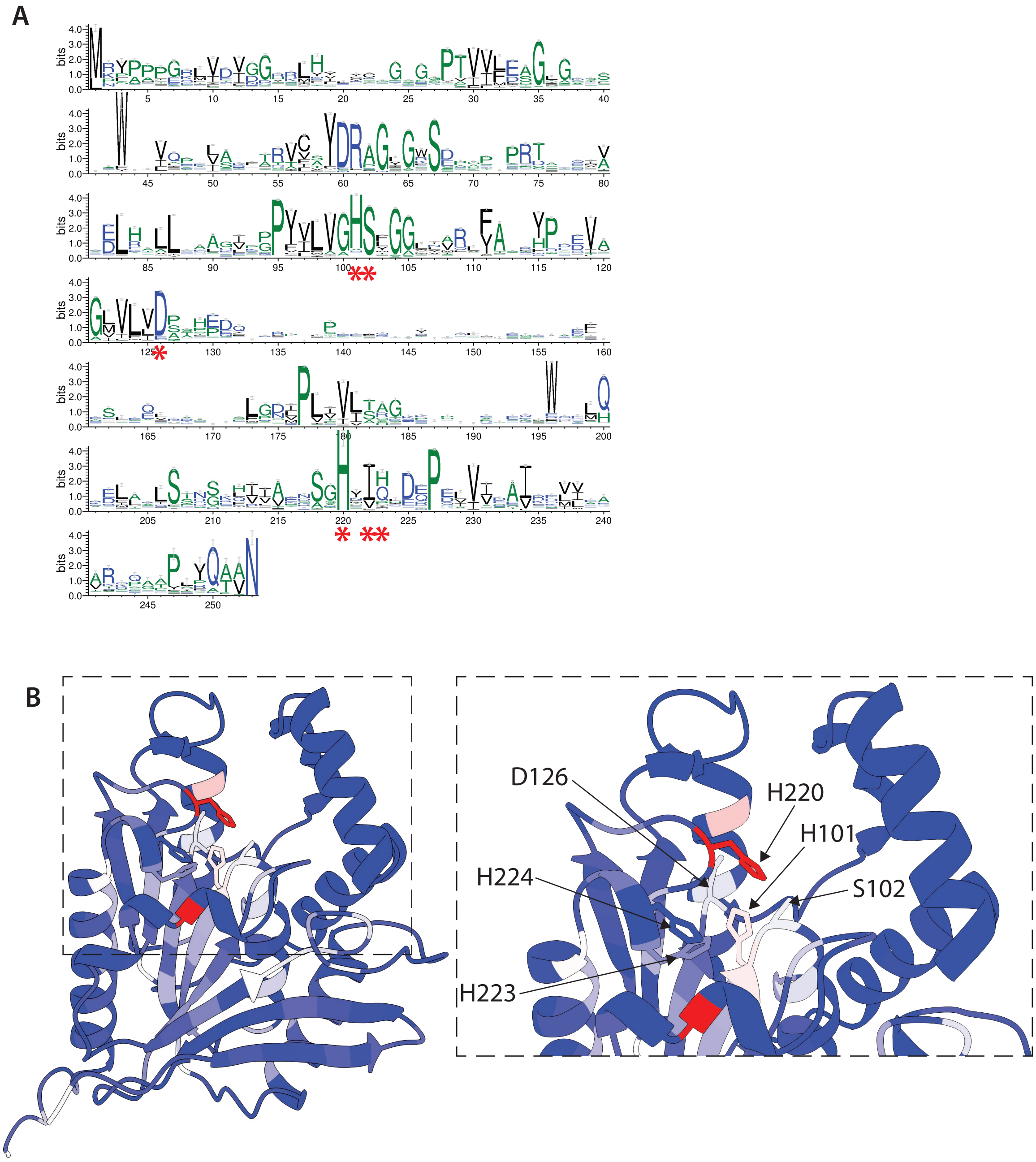

Figure S2. Conserved residues in UshA (YqjL) and their location within the AlphaFold-predicted struture. (A) WebLogo of UshA homologs. **(B)** AlphaFold-predicted structure with highly conserved residues highlighted in red and nonconserved residues in blue.

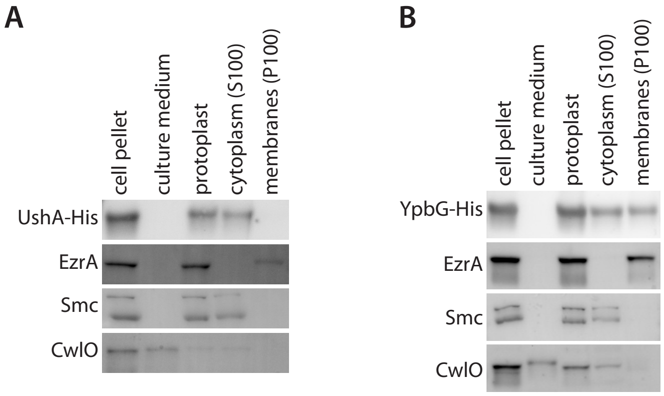

Figure S3. Subcellular fractionation of UshA-His and YpbG-His. Immunoblots of the indicated proteins after subcellular fractionation. **(A)** UshA-His was present in the soluble fraction of the protoplast lysate after ultracentrifugation indicating it is a cytoplasmic protein. (**B**) YpbG-His was present in soluble and membrane fractions. YpbG is predicted to have a N-terminal transmembrane helix, consistent with its presence in the pellet after ultracentrifugation. The presence of YpbG-His in the soluble fraction could reflect that YpbG is membrane-associated rather than an integral membrane protein. Alternatively, the protein in the soluble fraction could represent a proteolytic product that removes the TM segment but has similar mobility to the full-length protein. EzrA was analzyed as an integral membrane protein control. SMC was analyzed as a cytoplasmic protein control. CwlO was analyzed as a secreted protein control.

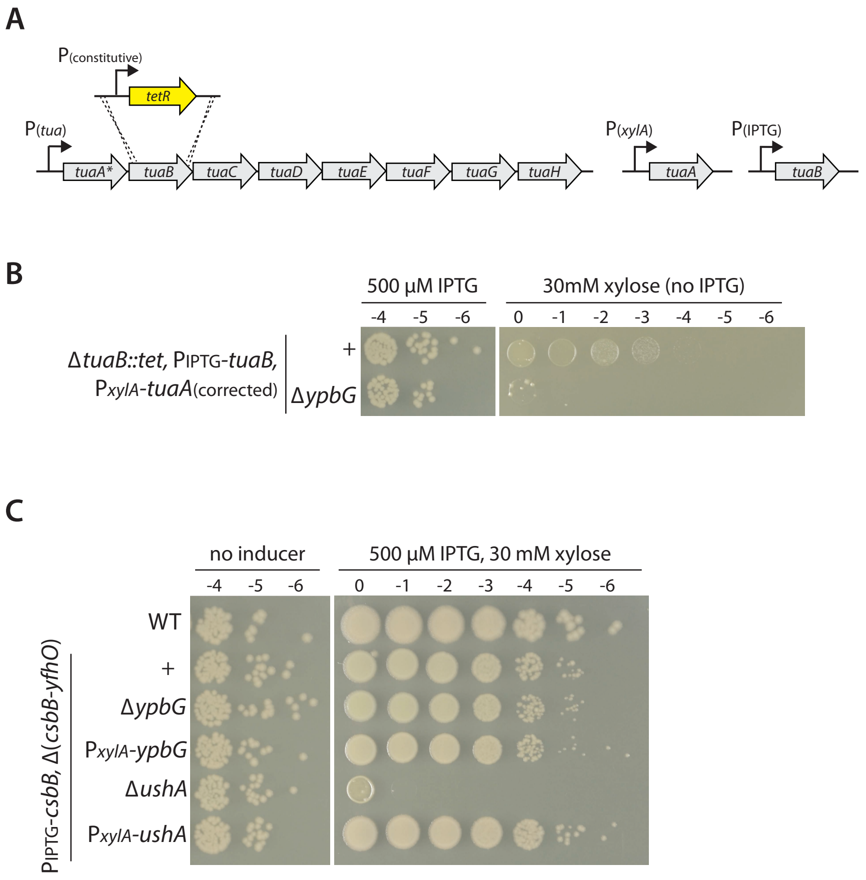

Figure S4. *ypbG* (*upsH*) becomes essential when UndP is trapped in the teichuronic acid biosynthesis pathway. (A) Schematic diagram of the strain used in this figure. In the *B. subtilis* PY79 strain, the *tuaA* gene is a psuedogene. An intact *tuaA* gene was fused to the xylose-regulated promoter P(*xylA*) and inserted at a non-essential locus in the genome. The *tuaB* gene encodes the transporter of the UndPP-linked TUA precursor. Its inactivation causes sequestration of lipid-linked precursors in the inner leaflet of the membrane. The *tetR* cassette constitutively expresses the other genes in the operon. The *tuaB* gene was fused to an IPTG-regulated promoter and inserted at a non-essential locus in the *B. subtilis* genome. **(B)** Photographs of spot-dilution assays of the indicated strains spotted on LB agar plates in the presence of 500 µM IPTG or 30 mM xylose. Cells expressing *tuaA* in the absence of TuaB are growth impaired but viable provided *ypbG* is intact. In the absence of *ypbG*, UndPP-TUA accumulation is toxic. **(C)** Photographs of spot-dilution assay of the indicated strains spotted on LB agar plates in the absence or presence of 500 µM IPTG and 30 mM xylose. Cells lacking or over-expressing *ypbG* have no impact on growth when UndP-linked sugars accumulate, consistent with the idea that YpbG acts specifically on UndPP-linked secondary cell wall polymer precursors. Δ*ushA* and over-expression of *ushA* serve as positive controls.

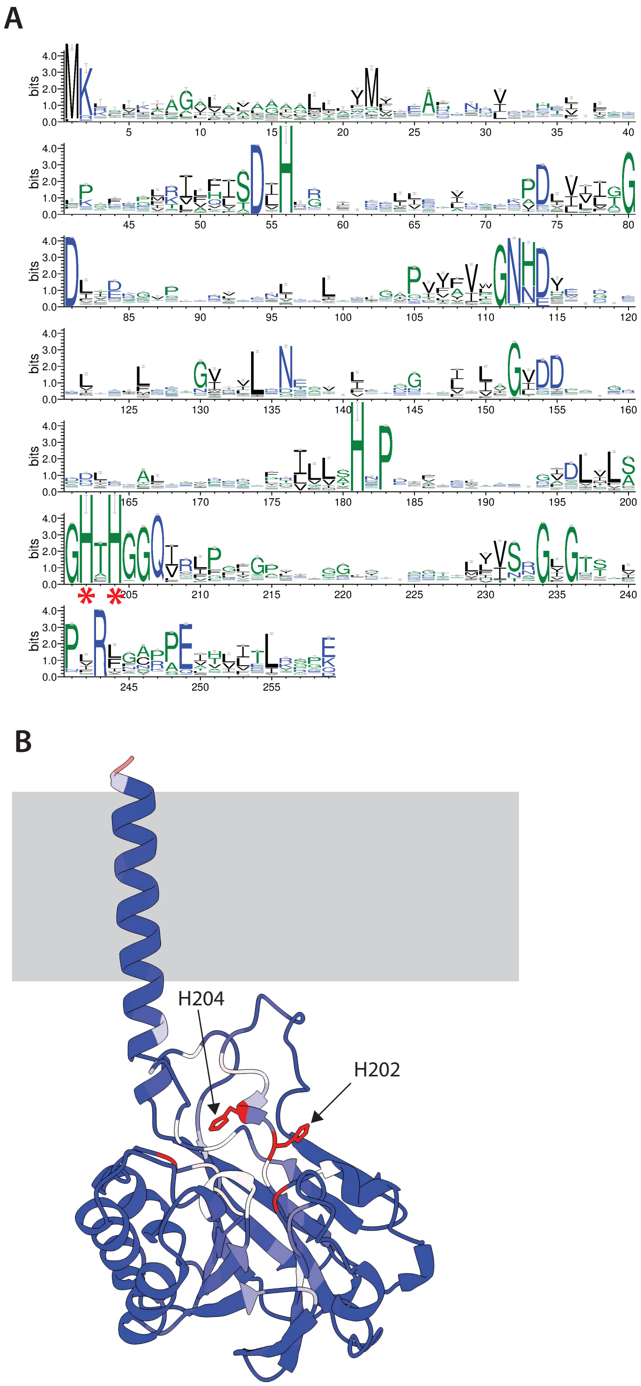

Figure S5. Conserved residues in YpbG (UpsH) and their location within the AlphaFold-predicted struture. (A) WebLogo of YpbG homologs. **(B)** AlphaFold-predicted structure with highly conserved residues highlighted in red and nonconserved residues in blue.

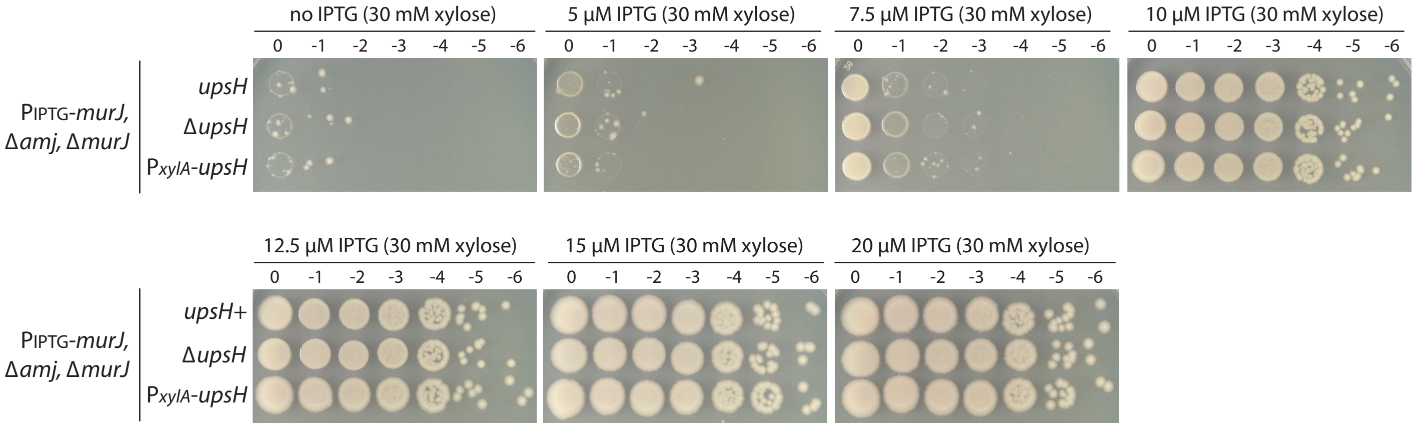

Figure S6. Deletion or overexpression of *upsH (ypbG)* does not enhance or suppress the growth defects associated with depletion of the lipid II flippase MurJ. Photographs of spot dilutions assays of the indicated strains on LB agar plates with 30 mM xylose and the indicated concentrations of IPTG. All three strains lack *murJ* and *amj* and harbor an IPTG-regulated allele of *murJ*. The *upsH*+ strain, the Δ*yupsH* mutant, and the strain overexpressing *upsH* are similarly growth impaired when MurJ becomes limiting. These data argue that UpsH does not act on the UndPP-linked muropeptide, lipid II.

SUPPLEMENTAL METHODS

Strain constructions

B. subtilis deletion mutants

Most *B. subtilis* deletion mutants were made by isothermal assembly (1) followed by direct transformation in *B. subtilis*. The assembly reactions contained three PCR products: two PCR products containing ~1500 base pairs upstream and downstream of the gene to be deleted, and a third PCR product containing an antibiotic resistance cassette. Antibiotic resistance cassettes with surrounding lox66/lox71 sites were amplified from pWX465(cat), pWX466(spec), pWX467(erm), pWX469(tet) and pWX470(kan) using the primers oJM028 and oJM029. The flanking regions for the respective deletions were amplified using PY79 genomic DNA as template and the following primer sets: *yngC*(oIR483-486); *tagG(oIR384-387); sigM-yhdL-yhdK*(oIR40,24,25,43); *ykoST*(oIR765-768); *csbB-yfhO*(oIR769,770,078,079); *ykcBC*(oIR761-764); *ggaAB*(oIR710,711,716,717); *ypbG*(oIR853-856); *yqjL*(oIR857-860); *ywnJ*(oIR861-864); *ycgR-ycgQ*(oIR866-oIR869); *yebC*(oIR870-873).

The *bcrC* deletion was from the BKE collection and was backcrossed twice into PY79 and PCR confirmed.

Construction of *yqjL-his10(ushA) and ypbG-his10(upsH)* point mutations

Point mutations in *yqjL-his10* and *ypbG-his10* were made by isothermal assembly and direct transformation into *B. subtilis*. Two DNA fragments were amplified using the genomic DNA of BIR1665 [yvbJ-PxylA-yqjL-his10-kan-yvbJ] or BIR1587 [yvbJ-PxylA -ypbG-his10-kan-yvbJ] as template using oligos flanking the upstream and downstream homology arms (oIR929 and oIR930) and mutation specific primers:
yqjL(H101A): oIR947 + oIR948
yqjL(S102A): oIR892 + oIR893
yqjL(D126A): oIR894 + oIR895
yqjL(H220A): oIR890 + oIR891
yqjL(H223A): oIR1085 + oIR1086
yqjL(H224A): oIR1087 + oIR1088
ypbG(H202A): oIR1420 + oIR1424
ypbG H204A): oIR1421 + oIR1424
ypbG(H202AH204A): oIR1422 + oIR1424

The two resulting amplification products were purified and added to an isothermal assembly reaction followed by direct transformation into BIR1050 or BIR1583:
BIR1050 [*sacA::Pveg-mTagBFP (phleo), amyE::Pamj-YFP (cat), ycgO::Phyperspank-optRBS-csbB (spec), yfhO::tet, yqjL::erm*)]
BIR1583 [*ycgO::Pspank*-tuaB (erm), tuaB::tet, amyE::Pamj-YFP (cat), sacA::Pveg-mTagBFP (phleo), ypbG::spec*].

All mutants were confirmed by sequencing.

<u>Plasmid Constructions</u>

pIR315 [yvbJ::PxylA-yqjL (kanR) (ampR)]
pIR315 was generated in a two-piece isothermal assembly reaction with PCR product containing the yqjL gene (amplified from PY79 gDNA with oIR874 and oIR875) and pCB133 [yvbJ::PxylA(kan)] digested with XhoI and BamHI.

pIR324 [yvbJ::PxylA-yqjL-his10 (kanR) (ampR)]
pIR324 was generated in a three-piece isothermal assembly reaction with PCR product containing the yqjL gene (amplified from PY79 gDNA with oIR886 and oIR887) and a his10 linker (amplified from pIR301-ycgO-Phyperspank-MCS-linker-his10 with oIR888 and oIR889) and linearized pCB133 [yvbJ::PxylA(kan)] (amplified with oIR680 and oIR681).

pIR439 [yvbJ::PxylA-ypbG (kanR) (ampR)]
pIR439 was generated in a two-piece isothermal assembly reaction with PCR product containing the ypbG gene (amplified from PY79 gDNA with oIR865 and oIR839) and pCB133 [yvbJ::PxylA(kan)] digested with XhoI and BamHI.

pIR476 [pLow-ypbG (ermR) (ampR)]
pIR476 was generated in a two-piece isothermal assembly reaction with PCR product containing the ypbG gene (amplified from PY79 gDNA with oIR1404 and oIR1405) and pLow digested with EcoRI and BamHI.

pIR333 [ycgO::Phyperspank-tuaA(corrected) (specR) (ampR)]
pIR333 was generated in a three-piece isothermal assembly reaction with PCR product containing the 5' end of the tuaA gene (amplified from PY79 gDNA with oIR791 and oIR917), the 3' end of the tuaA gene (amplified from PY79 gDNA with oIR792 and oIR916) and pCB090 [ycgO::Phyperspank(spec)] digested with HindIII and SpeI.

All plasmids were sequence-confirmed. All gene fusions to the Phyperspank promoter contained a synthetic optimized ribosome binding. All gene fusions to the Pspank promoter contained the native ribosome binding site.

References:

1. Gibson DG, Young L, Chuang R-Y, Venter JC, Hutchison CA, Smith HO. 2009. Enzymatic assembly of DNA molecules up to several hundred kilobases. Nat Methods 6:343–345.

Supplementary Table 1. Strains used in this study

Strain	Background	Genotype	Source	Figures
BIR003	*B. subtilis PY79*	*wildtype*	(1)	1c,1d,2f,2b,s1
BIR0334	*B. subtilis PY79*	*sacA::Pveg-mTagBFP (phleo), amyE::Pamj-YFP (cat)*	(2)	1be,3cde,4b,5bc,s4c
BIR0614	*B. subtilis PY79*	*sacA::Pveg-mTagBFP (phleo), amyE::Pamj-YFP (cat), ycgO::Pspank-tagG (spec), tagG::tet*	(2)	5b
BIR0616	*B. subtilis PY79*	*sacA::Pveg-mTagBFP (phleo), amyE::Pamj-YFP (cat), ycgO::Pspank-murJ (spec), murJ::tet, amj::erm*	(2)	s6
BIR0880	*B. subtilis PY79*	*ycgO::Pspank*-tuaB (erm), tuaB::tet, amyE::Pamj-YFP (cat), sacA::Pveg-mTagBFP (phleo)*	(2)	3cde,4b
BIR0894	*B. subtilis PY79*	*sacA::Pveg-mTagBFP (phleo), amyE::Pamj-YFP (cat), ycgO::Phyperspank-optRBS-ykcC (spec), ykcBC::tet*	(2)	2f
BIR0895	*B. subtilis PY79*	*sacA::Pveg-mTagBFP (phleo), amyE::Pamj-YFP (cat), ycgO::Phyper-optRBS-ggaA(spec), ggaAB::tet*	(2)	5c
BIR0901	*B. subtilis PY79*	*sacA::Pveg-mTagBFP (phleo), amyE::Pamj-YFP (cat), ycgO::Phyperspank-optRBS-csbB (spec), csbB-yfhO::erm*	(2)	1bcde,2bf,s4c
BIR0918	*B. subtilis PY79*	*sacA::Pveg-mTagBFP (phleo), amyE::Pamj-YFP (cat), ycgO::Phyperspank-optRBS-csbB (spec), csbB-yfhO::erm, mlk::kan*	(2)	1b
BIR0922	*B. subtilis PY79*	*sacA::Pveg-mTagBFP (phleo), amyE::Pamj-YFP (cat), ycgO::Phyperspank-optRBS-csbB (spec), csbB-yfhO::erm, mlk::tet*	(2)	1c
BIR0928	*B. subtilis PY79*	*sacA::Pveg-mTagBFP (phleo), amyE::Pamj-YFP (cat), ycgO::Phyper-optRBS-ggaA(spec), ggaAB::tet, ypbG::kan*	This work	5c
BIR0929	*B. subtilis PY79*	*sacA::Pveg-mTagBFP (phleo), amyE::Pamj-YFP (cat), ycgO::Phyperspank-optRBS-csbB (spec), csbB-yfhO::erm, ypbG::kan*	This work	1b,s4c
BIR0943	*B. subtilis PY79*	*sacA::Pveg-mTagBFP (phleo), amyE::Pamj-YFP, ycgO::Phyperspank-optRBS-ykcC (spec), ykcBC::tet, yqjL::Kan*	This work	2f
BIR0947	*B. subtilis PY79*	*sacA::Pveg-mTagBFP (phleo), amyE::Pamj-YFP (cat), ycgO::Phyperspank-optRBS-csbB (spec), csbB-yfhO::erm, yqjL::Kan*	This work	1bde
BIR0948	*B. subtilis PY79*	*sacA::Pveg-mTagBFP (phleo), amyE::Pamj-YFP (cat), ycgO::Phyper-optRBS-ggaA(spec), ggaAB::tet, yqjL::Kan*	This work	5c
BIR0988	*B. subtilis PY79*	*sacA::Pveg-mTagBFP (phleo), amyE::Pamj-YFP (cat), ycgO::Phyperspank-optRBS-csbB (spec), yfhO::tet, yqjL::kan*	This work	6a,s1
BIR1021	*B. subtilis PY79*	*sacA::Pveg-mTagBFP (phleo), amyE::Pamj-YFP (cat), ycgO::Phyperspank-optRBS-csbB (spec), yfhO::tet, yvbJ::PxylA-optRBS-yqjL (kan)*	This work	6a,s1
BIR1033	*B. subtilis PY79*	*sacA::Pveg-mTagBFP (phleo), amyE::Pamj-YFP (cat), ycgO::Phyperspank-optRBS-csbB (spec), csbB-yfhO::erm, ywnJ::Kan*	This work	1b
BIR1035	*B. subtilis PY79*	*sacA::Pveg-mTagBFP (phleo), amyE::Pamj-YFP (cat), ycgO::Phyperspank-optRBS-csbB (spec), csbB-yfhO::erm, yebC::kan*	This work	1b
BIR1036	*B. subtilis PY79*	*sacA::Pveg-mTagBFP (phleo), amyE::Pamj-YFP (cat), ycgO::Phyperspank-optRBS-csbB (spec), csbB-yfhO::erm, ycgQR::kan*	This work	1b
BIR1037	*B. subtilis PY79*	*sacA::Pveg-mTagBFP (phleo), amyE::Pamj-YFP (cat), ycgO::Phyperspank-optRBS-csbB (spec), csbB-yfhO::kan, bcrC::erm*	This work	1b
BIR1038	*B. subtilis PY79*	*sacA::Pveg-mTagBFP (phleo), amyE::Pamj-YFP (cat), ycgO::Phyperspank-optRBS-csbB (spec), csbB-yfhO::kan, yngC::erm*	This work	1b
BIR1079	*B. subtilis PY79*	*amyE::PxylA-gfp-spoIVFA (cat), yvbJ::PxylA-yqjL-his10 (kan)*	This work	2d, s3
BIR1099	*B. subtilis PY79*	*ycgO::Pspank*-tuaB (erm), tuaB::tet, amyE::Pamj-YFP (cat), sacA::Pveg-mTagBFP (phleo), mlk::kan*	This work	3c
BIR1159	*B. subtilis PY79*	*sacA::Pveg-mTagBFP (phleo), amyE::Pamj-YFP (cat), ycgO::Pspank-tagG (spec), tagG::tet, yqjL::kan*	This work	5b

BIR1228	*B. subtilis PY79*	*sacA::Pveg-mTagBFP (phleo), amyE::Pamj-YFP (cat), ycgO::Pspank-tagG (spec), tagG::tet, ypbG::kan*	This work	5b
BIR1241	*B. subtilis PY79*	*ycgO::Pspank*-tuaB (erm), tuaB::tet, amyE::Pamj-YFP (cat), sacA::Pveg-mTagBFP (phleo), yngC::kan*	This work	3c
BIR1242	*B. subtilis PY79*	*ycgO::Pspank*-tuaB (erm), tuaB::tet, amyE::Pamj-YFP (cat), sacA::Pveg-mTagBFP (phleo), ypbG::kan*	This work	3de,4b,6b,s1
BIR1243	*B. subtilis PY79*	*ycgO::Pspank*-tuaB (erm), tuaB::tet, amyE::Pamj-YFP (cat), sacA::Pveg-mTagBFP (phleo), ywnJ::kan*	This work	3c
BIR1244	*B. subtilis PY79*	*ycgO::Pspank*-tuaB (erm), tuaB::tet, amyE::Pamj-YFP (cat), sacA::Pveg-mTagBFP (phleo), yqjL::kan*	This work	3c
BIR1245	*B. subtilis PY79*	*ycgO::Pspank*-tuaB (erm), tuaB::tet, amyE::Pamj-YFP (cat), sacA::Pveg-mTagBFP (phleo), yebC::kan*	This work	3c
BIR1246	*B. subtilis PY79*	*ycgO::Pspank*-tuaB (erm), tuaB::tet, amyE::Pamj-YFP (cat), sacA::Pveg-mTagBFP (phleo), ycgQR::kan*	This work	3c
BIR1467	*S. aureus RN4220*	*pLow (ermR)*	(3)	5d
BIR1542	*B. subtilis PY79*	*sacA::Pveg-mTagBFP (phleo), amyE::Pamj-YFP (cat), ycgO::Pspank-tagG (spec), tagG::tet, yvbJ::PxylA-ypbG(kan)*	This work	5b
BIR1561	*B. subtilis PY79*	*sacA::Pveg-mTagBFP (phleo), amyE::Pamj-YFP (cat), ycgO::Phyper-optRBS-ggaA(spec), ggaAB::tet, yvbJ::PxylA-ypbG(kan)*	This work	5c
BIR1562	*B. subtilis PY79*	*sacA::Pveg-mTagBFP (phleo), amyE::Pamj-YFP (cat), ycgO::Phyper-optRBS-ggaA(spec), ggaAB::tet, yvbJ::PxylA-optRBS-yqjL (kan)*	This work	5c
BIR1566	*B. subtilis PY79*	*ycgO::Pspank*-tuaB (erm), tuaB::tet, amyE::Pamj-YFP (cat), sacA::Pveg-mTagBFP (phleo), yvbJ::PxylA-ypbG(kan)*	This work	6b,s1
BIR1570	*B. subtilis PY79*	*amyE::Phyperspank-tarGH(S.aureus)(spec),tagGH::cat, ypbG::kan*	This work	5e
BIR1571	*B. subtilis PY79*	*amyE::Phyperspank-tarGH(S.aureus)(spec),tagGH::cat, yvbJ::PxylA-ypbG(kan)*	This work	5e
BIR1578	*B. subtilis PY79*	*ycgO::Pspank*-tuaB (erm), tuaB::tet, amyE::Pamj-YFP (cat), sacA::Pveg-mTagBFP (phleo), yvbJ::PxylA-tuaA (corrected)(kan)*	This work	S4b
BIR1582	*B. subtilis PY79*	*ycgO::Pspank*-tuaB (erm), tuaB::tet, amyE::Pamj-YFP (cat), sacA::Pveg-mTagBFP (phleo), mlk::spec*	This work	3d
BIR1584	*B. subtilis PY79*	*ycgO::Pspank*-tuaB (erm), tuaB::tet, amyE::Pamj-YFP (cat), sacA::Pveg-mTagBFP (phleo), yvbJ::PxylA-ypbG(kan), mlk::spec*	This work	3d
BIR1585	*B. subtilis PY79*	*ycgO::Pspank*-tuaB (erm), tuaB::tet, amyE::Pamj-YFP (cat), sacA::Pveg-mTagBFP (phleo), yvbJ::PxylA-ypbG(kan), ypbG::spec*	This work	3d,4bc
BIR1587	*B. subtilis PY79*	*ycgO::Pspank*-tuaB (erm), tuaB::tet, amyE::Pamj-YFP (cat), sacA::Pveg-mTagBFP (phleo), yvbJ::PxylA-ypbG-his10(kan), ypbG::spec*	This work	4bc
BIR1589	*B. subtilis PY79*	*ycgO::Pspank*-tuaB (erm), tuaB::tet, amyE::Pamj-YFP (cat), sacA::Pveg-mTagBFP (phleo), yvbJ::PxylA-tuaA (corrected)(kan), ypbG::spec*	This work	S4b
BIR1591	*B. subtilis PY79*	*amyE::PxylA-gfp-spoIVFA (cat), yvbJ::PxylA-ypbG-his10(kan)*	This work	4d,s3
BIR1599	*B. subtilis PY79*	*ycgO::Pspank*-tuaB (erm), tuaB::tet, amyE::Pamj-YFP (cat), sacA::Pveg-mTagBFP (phleo), yvbJ::PxylA-ypbG-his10(H202A)(kan), ypbG::spec*	This work	4bc
BIR1600	*B. subtilis PY79*	*ycgO::Pspank*-tuaB (erm), tuaB::tet, amyE::Pamj-YFP (cat), sacA::Pveg-mTagBFP (phleo), yvbJ::PxylA-ypbG-his10(H204A)(kan), ypbG::spec*	This work	4bc
BIR1601	*B. subtilis PY79*	*ycgO::Pspank*-tuaB (erm), tuaB::tet, amyE::Pamj-YFP (cat), sacA::Pveg-mTagBFP (phleo), yvbJ::PxylA-ypbG-his10(H202A,H204A)(kan), ypbG::spec*	This work	4bc
BIR1637	*B. subtilis PY79*	*sacA::Pveg-mTagBFP (phleo), amyE::Pamj-YFP (cat), ycgO::Phyperspank-optRBS-csbB (spec), csbB-yfhO::erm, yvbJ::PxylA-yqjL (kan)*	This work	s4c

BIR1638	*B. subtilis PY79*	*sacA::Pveg-mTagBFP (phleo), amyE::Pamj-YFP (cat), ycgO::Phyperspank-optRBS-csbB (spec), csbB-yfhO::erm, yvbJ::PxylA-ypbG (kan)*	This work	s4c
BIR1648	*S. aureus RN4220*	*pLow-ypbG (ermR)*	This work	5d
BIR1649	*B. subtilis PY79*	*sacA::Pveg-mTagBFP (phleo), amyE::Pamj-YFP (cat), ycgO::Phyperspank-optRBS-csbB (spec), csbB, yfhO::erm, yqjL::tet*	This work	1c,2bf,s4c
BIR1651	*B. subtilis PY79*	*sacA::Pveg-mTagBFP (phleo), amyE::Pamj-YFP (cat), ycgO::Phyperspank-optRBS-ykoT (spec), ykoS-ykoT::erm*	This work	2f
BIR1653	*B. subtilis PY79*	*sacA::Pveg-mTagBFP (phleo), amyE::Pamj-YFP (cat), ycgO::Phyperspank-optRBS-csbB (spec), csbB-yfhO::erm, mlk::tet, yvbJ::PxylA-optRBS-yqjL (kan)*	This work	1c
BIR1664	*B. subtilis PY79*	*sacA::Pveg-mTagBFP (phleo), amyE::Pamj-YFP (cat), ycgO::Phyperspank-optRBS-csbB (spec), csbB-yfhO::erm, yqjL::tet, yvbJ::PxylA-optRBS-yqjL (kan)*	This work	1c,2bc
BIR1665	*B. subtilis PY79*	*ycgO::Phyperspank-optRBS-csbB (spec), csbB-yfhO::erm, yqjL::tet, yvbJ::PxylA-optRBS-yqjL-his10 (kan)*	This work	2bc
BIR1666	*B. subtilis PY79*	*ycgO::Phyperspank-optRBS-csbB (spec), csbB-yfhO::erm, yqjL::tet, yvbJ::PxylA-optRBS-yqjL(H101A)-his10 (kan)*	This work	2bc
BIR1667	*B. subtilis PY79*	*ycgO::Phyperspank-optRBS-csbB (spec), csbB-yfhO::erm, yqjL::tet, yvbJ::PxylA-optRBS-yqjL(S102A)-his10 (kan)*	This work	2bc
BIR1668	*B. subtilis PY79*	*ycgO::Phyperspank-optRBS-csbB (spec), csbB-yfhO::erm, yqjL::tet, yvbJ::PxylA-optRBS-yqjL(D126A)-his10 (kan)*	This work	2bc
BIR1669	*B. subtilis PY79*	*ycgO::Phyperspank-optRBS-csbB (spec), csbB-yfhO::erm, yqjL::tet, yvbJ::PxylA-optRBS-yqjL(H220A)-his10 (kan)*	This work	2bc
BIR1670	*B. subtilis PY79*	*ycgO::Phyperspank-optRBS-csbB (spec), csbB-yfhO::erm, yqjL::tet, yvbJ::PxylA-optRBS-yqjL(H223A)-his10 (kan)*	This work	2bc
BIR1671	*B. subtilis PY79*	*ycgO::Phyperspank-optRBS-csbB (spec), csbB-yfhO::erm, yqjL::tet, yvbJ::PxylA-optRBS-yqjL(H224A)-his10 (kan)*	This work	2bc
BIR1674	*B. subtilis PY79*	*sacA::Pveg-mTagBFP (phleo), amyE::Pamj-YFP (cat), ycgO::Phyperspank-optRBS-ykoT (spec), ykoS-ykoT::erm, yqjL::kan*	This work	2f
BIR1677	*B. subtilis PY79*	*sacA::Pveg-mTagBFP (phleo), amyE::Pamj-YFP (cat), ycgO::Pspank-murJ (spec), murJ::tet, amj::erm, ypbG::kan*	This work	s6
BIR1679	*B. subtilis PY79*	*sacA::Pveg-mTagBFP (phleo), amyE::Pamj-YFP (cat), ycgO::Pspank-murJ (spec), murJ::tet, amj::erm, yvbJ::Pxyl-ypbG(kan)*	This work	s6

References:

1. Youngman P, Perkins JB, Losick R. 1984. Construction of a cloning site near one end of Tn917 into which foreign DNA may be inserted without affecting transposition in Bacillus subtilis or expression of the transposon-borne erm gene. Plasmid 12:1–9.

2. Roney IJ, Rudner DZ. 2024. Bacillus subtilis uses the SigM signaling pathway to prioritize the use of its lipid carrier for cell wall synthesis. PLoS Biol 22:e3002589.

3. Roney IJ, Rudner DZ. 2023. Two broadly conserved families of polyprenyl-phosphate transporters. Nature 613:729–734.

Supplementary Table 2. Plasmids used in this study

Plasmid	Description	Source
pAM155	amyE::Pamj-yfp(cat)(amp)	(1)
pIR175	ycgO::Phyperspank-csbB(spec) (amp)	(2)
pIR190	ycgO::Pspank-tagG(spec) (amp)	(2)
pIR192	ycgO::Pspank-murJ(spec) (amp)	(2)
pIR286	ycgO::Phyperspank-ykoT(spec) (amp)	(2)
pIR287	ycgO::Phyperspank-ykcC(spec) (amp)	(2)
pIR288	ycgO::Phyperspank-ggaA(spec) (amp)	(2)
pIR315	yvbJ::PxylA-yqjL(kan)(amp)	This paper
pIR324	yvbJ::PxylA-yqjL-his10 (kan)(amp)	This paper
pIR333	*ycgO::Phyperspank-tuaA(corrected)(specR)*	This paper
pIR439	yvbJ::PxylA-ypbG(kan)(amp)	This paper
pIR476	*pLow-ypbG (ermR)*	This paper

References:

1. Meeske AJ, Sham L-T, Kimsey H, Koo B-M, Gross CA, Bernhardt TG, Rudner DZ. 2015. MurJ and a novel lipid II flippase are required for cell wall biogenesis in *Bacillus subtilis*. Proc Natl Acad Sci USA 112:6437–6442.
2. Roney IJ, Rudner DZ. 2024. Bacillus subtilis uses the SigM signaling pathway to prioritize the use of its lipid carrier for cell wall synthesis. PLoS Biol 22:e3002589.

Supplementary Table 3. Oligonucleotides used in this study

Primer	Sequence
oIR0024	GGTACTGAGCGAGGGAGCAGAATGCCTTTTCTCCCCTCTATGTTATAC
oIR0025	CGGTAGTTGACCAGTGCTCCCTGAAAAGACGCCTTTTCAGGC
oIR0040	CTTCAGAAACGAACCGATCC
oIR0043	GATCGGTTCCATGAGTTCAGG
oIR0078	GTTGACCAGTGCTCCCTGAGCCGAGCTTTAATTTTTTCTG
oIR0079	CCAAAATCTTTCTCGTCTGG
oIR0384	ctcaattgattcagaacacc
oIR0385	CGGTACTGAGCGAGGGAGCAGAAtttttatcttccttagacttaattgtttttg
oIR0386	CGGTAGTTGACCAGTGCTCCCTGtacgtaaggagattttacgatg
oIR0387	gatgagccgtttgcgtttctg
oIR0483	gcagctcaataataaaactagaatcc
oIR0484	CGGTACTGAGCGAGGGAGCAGAAattcttcacaacctgtcctaatc
oIR0485	CGGTAGTTGACCAGTGCTCCCTGctagcggatatgcataggggtgac
oIR0486	cctgtcggcattgttgcaaac
oIR0487	cagaaagatcatagcctttgtcatg
oIR0680	CTCGAGatGCTAGCtcAAGCTTcattcaaat
oIR0681	GGATCccagcgaaccatttgaggtgatagg
oIR0710	GATTTTCGTTTATATCATATCAACCC
oIR0711	CGGTACTGAGCGAGGGAGCAGAAGTTTGTACCTCTTTATTTAGAAGTTAAAGG
oIR0716	CGGTAGTTGACCAGTGCTCCCTGGGTTGGTTTGTTTTATATTGACACTTC
oIR0717	GAATAGTTTAACCATAAATTTTTTCGATC
oIR0761	CGTACACTTCTTCAAGGTACGTATAAAGC
oIR0762	CGGTACTGAGCGAGGGAGCAGAATTATTTTTCACTCCTTTTTGTCTAACTTTGAAATAG
oIR0763	CGGTAGTTGACCAGTGCTCCCTGCCTCAAACCCCCTGTCCGTAATG
oIR0764	GATGAAAACAGAACGAAAGGTAATGAG
oIR0765	GCCACGGAGGACAATTTTTCTAACC
oIR0766	CGGTACTGAGCGAGGGAGCAGAATGCTTACACATCCATTTGTTATTCTG
oIR0767	CGGTAGTTGACCAGTGCTCCCTGACACGGAAAGAGCTGACTTCATTAG
oIR0768	CACATCATAGCGCATGGCGTTTAC
oIR0769	CATAAAAGCAGGAAAGCTGAATGTC
oIR0770	GGTACTGAGCGAGGGAGCAGAATAAGGCACCTTCTTTTTATTATTCTTTTTAAGTATTGC
oIR0791	AATTGTGAGCGGATAACAATTAAGCTTacataaggaggaactactATGAGTGCAGAGAAAAGCATGAATG
oIR0792	CgaGCTAGCatCTGCAGttACTAGTTTATCTTGCACCATCACCCGTCC
oIR0839	cctatcacctcaaatggttcgctggGATCCTTATTCTGGCCCGCAAAGAGTC
oIR0853	GTTATATTTGACGCAGCTACTCATC

oIR0854	CGGTACTGAGCGAGGGAGCAGAACTCTCCATTCTTTTTAGAACTTATCAATAAG
oIR0855	CGGTAGTTGACCAGTGCTCCCTGCTTCTAAAAAGCAAAAATCCGTATG
oIR0856	GGAACGAGAATATCACAATCCAGC
oIR0857	CAATCATGCCTCTTGAATTCACTTC
oIR0858	CGGTACTGAGCGAGGGAGCAGAATGGAGTCATCTCCTCTAATTGG
oIR0859	CGGTAGTTGACCAGTGCTCCCTGCGTAAAAAAAGACCGGGCCGTAAGG
oIR0860	CAATCGTTCCGAAGAATTCAGGAG
oIR0861	CATCAAAACAGACAGAGTGACAAG
oIR0862	CGGTACTGAGCGAGGGAGCAGAAGACATAACCTCCTTTATAACGTACG
oIR0863	CGGTAGTTGACCAGTGCTCCCTGAGCCGGCTGTCTTGATTTCAGAC
oIR0864	CTCATTTACACCTTCTTAGGAGGAG
oIR0865	TAGCatCTCGAGacataaggaggaactactATGAAGCTATCAGTGAAAATTGC
oIR0866	CAGACTCAGTATGACAAACGGTCAC
oIR0867	CGGTACTGAGCGAGGGAGCAGAAAATAAAACCTCCGCTCATGTTAAG
oIR0868	CGGTAGTTGACCAGTGCTCCCTGGTGAAGACGAAACCAGTACAAG
oIR0869	CTCAGCTTCAGATAAAGAACTGGTG
oIR0870	GCGACTATTGTTCGCTTTTTGTATTG
oIR0871	CGGTACTGAGCGAGGGAGCAGAAGTCAGCAACTCCTATCAAAAAAATACGG
oIR0872	CGGTAGTTGACCAGTGCTCCCTGTGAAAAAAACCGGCTAATCCTAGCC
oIR0873	CATTTGGACGATGGAAAGAAGTG
oIR0874	TAGCatCTCGAGacataaggaggaactactATGAAATCAGCTTGGATGGAAAAG
oIR0875	tcgctggGATCCTTAATTGACTGCCTGGTAAAGAGG
oIR0886	atgaaataaaatgcatctgtatttgaatg
oIR0887	atgaaataaaatgcatctgtatttgaatg
oIR0888	CCTCTTTACCAGGCAGTCAATACTAGTggcAGCGGCTCTcatc
oIR0889	cacctcaaatggttcgctggGATCCGTTTCCACCGAATTAGCTTGCATG
oIR0890	GGATTCAAGCTAAAAACAGCTCCGCTAACATCCATCATGATGAACCTC
oIR0891	GAGGTTCATCATGATGGATGTTAGCGGAGCTGTTTTTAGCTTGAATCC
oIR0892	CTTATTTGGCTGTTTCACACGCTTACGGAGCTGTCATCACCGGTTTATG
oIR0893	CCGGTGATGACAGCTCCGTAAGCGTGTGAAACAGCCAAATAAGG
oIR0894	TTATCGGCATGGTCCTTCTTGCTCCAGCTTTAGGCGATTGCGCCAGC
oIR0895	GCGCAATCGCCTAAAGCTGGAGCAAGAAGGACCATGCCGATAATATC
oIR0916	CTGTTAAACCGGGATTGAGCGGCTGGGCTCAGGTGAACGGCGGTTAC
oIR0917	CCAGCCGCTCAATCCCGGTTTAACAGCCAGACGCTGTGTAAAGCCTG
oIR0929	CAATCATTACGATGGTTCTTTTCAG
oIR0930	GTTCTGGTGAAACTGAAGACAGCAC
oIR0947	CTCCTTATTTGGCTGTTTCAGCATCATACGGAGCTGTCATCACCG

oIR0948	CGGTGATGACAGCTCCGTATGATGCTGAAACAGCCAAATAAGGAGG
oIR1085	GCTAAAAACAGCTCCCACAACATCGGACATGATGAACCTCATATCGTTCAC
oIR1086	GTGAACGATATGAGGTTCATCATGTCCGATGTTGTGGGAGCTGTTTTTAGC
oIR1087	CTAAAAACAGCTCCCACAACATCCATGGAGATGAACCTCATATCGTTCACTTG
oIR1088	CAAGTGAACGATATGAGGTTCATCTCCATGGATGTTGTGGGAGCTGTTTTTAG
oIR1404	tctagaGGATCCacataaggaggaactactATGAAG
oIR1405	gccagtGAATTCTTATTCTGGCCCGCAAAGAGTC
oIR1420	GATGACGGTATTGATGTGATACTCAGCGGAGCAACCCATGGAGGCCAGATCAGG
oIR1421	GATGACGGTATTGATGTGATACTCAGCGGACATACCGCAGGAGGCCAGATCAGGTTTGGAAAATTC
oIR1422	GATGACGGTATTGATGTGATACTCAGCGGAGCAACCGCAGGAGGCCAGATCAGGTTTGGAAAATTC
oIR1424	TCCGCTGAGTATCACATCAATAC
oJM028	TTCTGCTCCCTCGCTCAG
oJM029	CAGGGAGCACTGGTCAAC
